# *Yersinia pseudotuberculosis* YopJ Limits Macrophage Response by Downregulating COX-2-Mediated Biosynthesis of PGE2 in a MAPK/ERK-Dependent Manner

**DOI:** 10.1128/spectrum.00496-21

**Published:** 2021-07-28

**Authors:** Austin E. F. Sheppe, John Santelices, Daniel M. Czyz, Mariola J. Edelmann

**Affiliations:** a Department of Microbiology and Cell Science, College of Agricultural and Life Sciences, University of Florida, Gainesville, Florida, USA

**Keywords:** Gram-negative infection, *Yersinia*, eicosanoids, prostaglandin, proteomics

## Abstract

Prostaglandin E2 (PGE2) is an essential immunomodulatory lipid released by cells in response to infection with many bacteria, yet its function in macrophage-mediated bacterial clearance is poorly understood. *Yersinia* overall inhibits the inflammatory circuit, but its effect on PGE2 production is unknown. We hypothesized that one of the *Yersinia* effector proteins is responsible for the inhibition of PGE2 biosynthesis. We identified that *yopB*-deficient Y. enterocolitica and Y. pseudotuberculosis deficient in the secretion of virulence proteins via a type 3 secretion system (T3SS) failed to inhibit PGE2 biosynthesis in macrophages. Consistently, COX-2-mediated PGE2 biosynthesis is upregulated in cells treated with heat-killed or T3SS-deficient Y. pseudotuberculosis but diminished in the presence of a MAPK/ERK inhibitor. Mutants expressing catalytically inactive YopJ induce similar levels of PGE2 as heat-killed or Δ*yopB* Y. pseudotuberculosis, reversed by YopJ complementation. Shotgun proteomics discovered host pathways regulated in a YopJ-mediated manner, including pathways regulating PGE2 synthesis and oxidative phosphorylation. Consequently, this study identified that YopJ-mediated inhibition of MAPK signal transduction serves as a mechanism targeting PGE2, an alternative means of inflammasome inhibition by *Yersinia.* Finally, we showed that EP4 signaling supports macrophage function in clearing intracellular bacteria. In summary, our unique contribution was to determine a bacterial virulence factor that targets COX-2 transcription, thereby enhancing the intracellular survival of yersiniae. Future studies should investigate whether PGE2 or its stable synthetic derivatives could serve as a potential therapeutic molecule to improve the outcomes of specific bacterial infections. Since other pathogens encode YopJ homologs, this mechanism is expected to be present in other infections.

**IMPORTANCE** PGE2 is a critical immunomodulatory lipid, but its role in bacterial infection and pathogen clearance is poorly understood. We previously demonstrated that PGE2 leads to macrophage polarization toward the M1 phenotype and stimulates inflammasome activation in infected macrophages. Finally, we also discovered that PGE2 improved the clearance of Y. enterocolitica. The fact that Y. enterocolitica hampers PGE2 secretion in a type 3 secretion system (T3SS)-dependent manner and because PGE2 appears to assist macrophage in the clearance of this bacterium indicates that targeting of the eicosanoid pathway by *Yersinia* might be an adaption used to counteract host defenses. Our study identified a mechanism used by *Yersinia* that obstructs PGE2 biosynthesis in human macrophages. We showed that Y. pseudotuberculosis interferes with PGE2 biosynthesis by using one of its T3SS effectors, YopJ. Specifically, YopJ targets the host COX-2 enzyme responsible for PGE2 biosynthesis, which happens in a MAPK/ER-dependent manner. Moreover, in a shotgun proteomics study, we also discovered other pathways that catalytically active YopJ targets in the infected macrophages. YopJ was revealed to play a role in limiting host LPS responses, including repression of EGR1 and JUN proteins, which control transcriptional activation of proinflammatory cytokine production such as interleukin-1β. Since YopJ has homologs in other bacterial species, there are likely other pathogens that target and inhibit PGE2 biosynthesis. In summary, our study’s unique contribution was to determine a bacterial virulence factor that targets COX-2 transcription. Future studies should investigate whether PGE2 or its stable synthetic derivatives could serve as a potential therapeutic target.

## INTRODUCTION

Nonsteroidal anti-inflammatory drugs (NSAIDs) are compounds that primarily target proinflammatory prostaglandin production (1–3), and these medicines are taken to alleviate the symptoms of both viral and bacterial infections. NSAIDs typically act on cyclooxygenase (COX)-1/-2 enzymes, where COX-1/2 biosynthesize prostaglandins (PGs) that belong to the group of eicosanoids. The recent controversy surrounding ibuprofen and COVID-19 (4) further emphasizes that PGs’ function in infections and their regulation by NSAIDs has to be considered. PGs and leukotrienes (LTs) are the two major classes of eicosanoids and are the products of oxidation reactions involving cPLA2 liberated arachidonic acid (AA) and COX or lipoxygenase (LOX) enzymes, respectively (5, 6). PGE2 is the most abundantly produced PG, and it is rapidly generated via the conversion of free AA to PGH2 by COX-1/2 enzymes in response to lipopolysaccharide/Toll-like receptor 4 (LPS-TLR4) binding (7). COX-1 is constitutively expressed, while COX-2 is inducible by TLR signaling (8–10). PGE2 is secreted into the extracellular environment by multidrug resistance-associated protein (MRP) MRP4 and acts on distinct membrane-bound E-type prostanoid receptors (EP1 to EP4) on the cell surface (11). EP signaling induces changes in secondary messenger signals such as cAMP and calcium influx, resulting in gene transcription changes, contributing to the macrophage polarization toward proinflammatory M1 phenotype (12, 13).

The functions of PGE2 in specific bacterial infections remain largely unknown (14), and the pathogenic mechanisms that lead to the perturbations of this lipid metabolism in the host cell also need to be further explored. PGE2 has complex functions in inflammation. For example, prostaglandins often amplify the production of interleukin-1β (IL-1β), a cytokine vital to the function of the inflammasome (15–18). Specifically, IL-1β synthesis has been previously demonstrated to require functional prostaglandin E_2_ (PGE2) biosynthesis in macrophages (17), and this observation has been supported in other studies in human and murine macrophages (13, 19). In contrast, in a cryopyrin-associated periodic syndrome model, PGE2 inhibits inflammasome activation by binding to EP4 receptors (CAPS) (20). Hence, the relationship between the PGE2 and IL-1β cannot be expected to be the same in various infection models and warrants further investigation (14). PGs are generated in response to infections with many Gram-negative pathogens, including Salmonella enterica, Escherichia coli, Chlamydia trachomatis, and Legionella pneumophila (8, 9, 13, 21, 22), as well as the acid-fast bacterium Mycobacterium tuberculosis (23, 24) and the Gram-positive bacterium Staphylococcus aureus (22, 25). However, our group has identified that at least one Gram-negative bacterium, *Yersinia* subsp., has a likely mechanism to counteract PGE2 synthesis, which for now remains unknown and the effect on PGE2 itself has not been confirmed.

The family of *Yersiniaceae* encompasses three Gram-negative pathogens Yersinia enterocolitica, Y. pseudotuberculosis, and Y. pestis. While Y. pestis is widely known for three plague events (26, 27) and is still responsible for minor outbreaks (28–30), Y. pseudotuberculosis and Y. enterocolitica infections cause a significant gastrointestinal disease known as yersiniosis, which causes inflammation, fever, bloody diarrhea, and swelling of the lymph nodes (31). All three yersiniae contain a conserved pYV virulence plasmid, which aids in establishing infection inside the host (32, 33) via encoded Y*ersinia* outer proteins (Yops) and a type 3 secretion system (T3SS). T3SS translocates Yops into host immune cells such as macrophages to alter immune cell functions, including inflammatory cytokine production (34–39). As one example, yersiniae dampen the inflammasome response in host macrophages mediated by specific Yop effectors such as YopJ and YopM (40, 41). We have previously demonstrated that Y. enterocolitica inhibits IL-1β production by downregulating PGE2 biosynthesis from THP-1 macrophages in a pYV-dependent manner (13). We also showed that PGE2 significantly reduced bacterial survival in macrophages (13), indicating that PGE2 downregulation might be an important aspect of Y. enterocolitica pathogenesis. However, the pYV-deficient Y. enterocolitica strain has a pleiotropic change in virulence and cellular outcomes, and COX-1/2 required for PGE2 production is regulated transcriptionally and posttranscriptionally (reviewed in reference 42). Hence, in the past study, we were unable to determine a specific mechanism by which *Yersinia* alters PGE2 levels, and the mechanism behind Y. enterocolitica inhibition of PGE2 remained unclear.

Here, we hypothesized that yersiniae use specific effector Yops to downregulate PGE2 and that this PGE2 downregulation is essential in the survival of this bacterium within macrophages. Given the importance of PGE2 signaling during infection with Y. enterocolitica, this study aimed to identify the molecular mechanism behind the *Yersinia*-mediated inhibition of PGE2 during macrophage infection with Y. enterocolitica and Y. pseudotuberculosis. Of particular interest is the YopJ virulence factor, which inhibits several mitogen-activated protein kinases (MAPKs), including the MAPK/ERK (MEK) pathway (36, 43), which plays a role in the cytokine transcription activation via NF-κB or c-JUN/AP-1 (43–45). This study identified YopJ-mediated downregulation of LPS signaling as a mechanism behind *Yersinia*-mediated inhibition of PGE2 biosynthesis in infected human macrophages. Understanding the role of PGE2 during bacterial infection can support future studies focused on the investigation of PGE2 or its stable synthetic derivatives as potential therapeutic molecules to improve the outcomes of specific bacterial infections.

## RESULTS

### PGE2 and IL-1β biosynthesis is altered in response to Δ*yopB* Y. enterocolitica*-* and Y. pseudotuberculosis*-*infected THP-1 macrophages.

**FIG 1:**
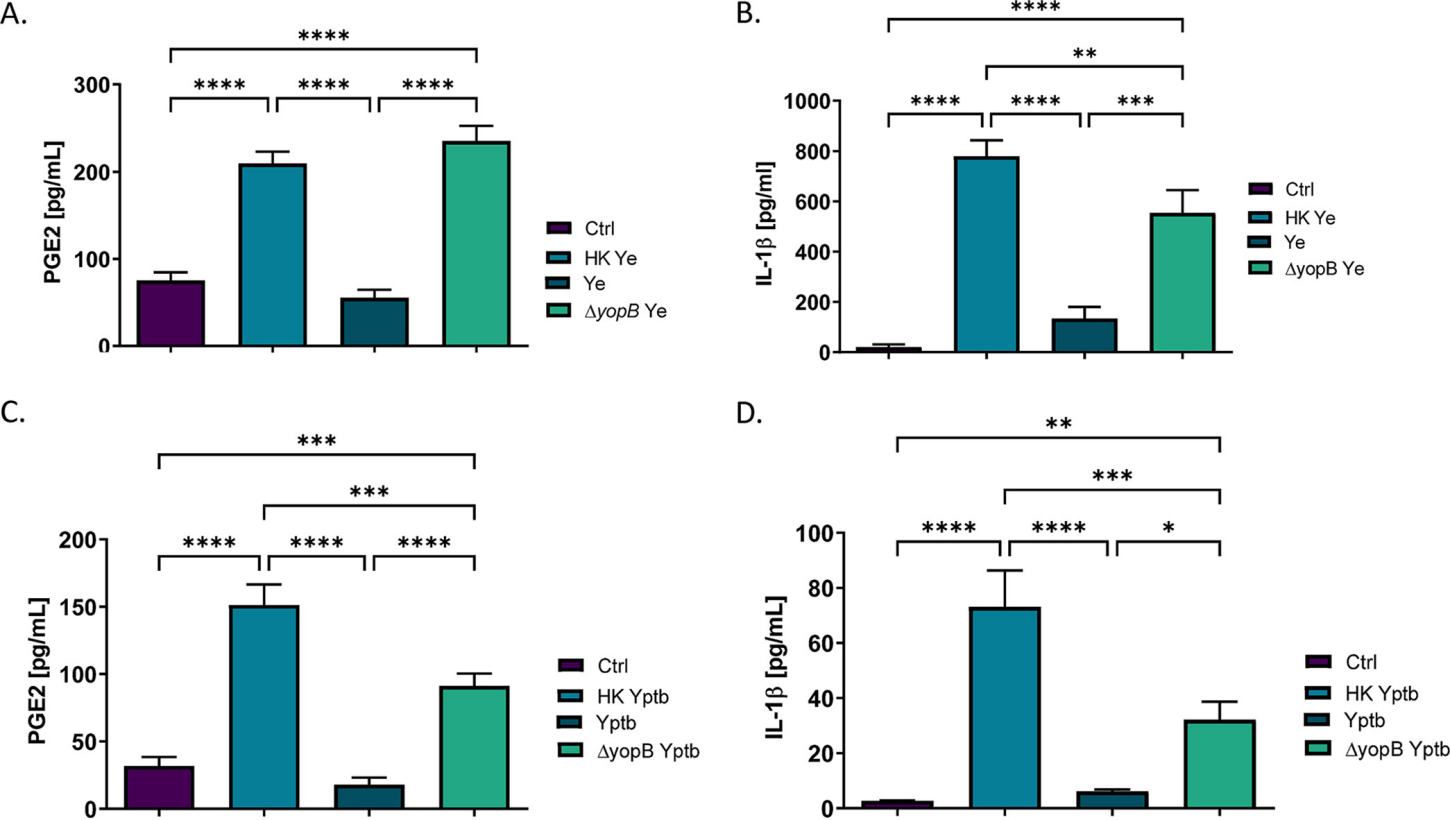
Analysis of PGE2 and IL-1β secretion in THP-1 macrophages infected with wild-type, *ΔyopB*, or heat-killed Y. enterocolitica and Y. pseudotuberculosis. PMA-differentiated THP-1 macrophages were infected at an MOI of 50:1 with wild type, Δ*yopB* Y. enterocolitica (Ye; A and B) and Y. pseudotuberculosis (Yptb; C and D) or treated with an equivalent number of heat-killed bacteria for 2 h. Uninfected macrophages were used as a control (Ctrl). Bacteria were heat killed at 55°C for 30 min before treating macrophages, and loss of viability was confirmed by plating. Commercial monoclonal ELISAs were used to measure the concentrations of PGE2 (A and C) and IL-1β (B and D) in supernatants from Y. enterocolitica- or Y. pseudotuberculosis-infected THP-1 cells. One-way ANOVA and Tukey’s *post hoc* test were used to calculate significance (*n* = 3). *P* values are indicated (*, *P* ≤ 0.05; **, *P* ≤ 0.01; ***, *P* ≤ 0.001; ****, *P* ≤ 0.0001). The data are representative of three independent experiments.

To experimentally determine whether YopJ affects COX-2 biosynthesis, we hypothesized that wild-type Y. enterocolitica or wild-type Y. pseudotuberculosis requires a functional T3SS to restrict biosynthesis and secretion of PGE2 from THP-1 macrophages. It was important to determine whether heat-killed Y. enterocolitica induces PGE2 secretion compared to viable bacteria since heat inactivation stops novel protein synthesis by damaging essential ribosomes, as well as by releasing cellular components by damaging the bacterial outer membrane. Moreover, to test whether translocation of bacterial effectors contributes to hinderance of host PGE2 synthesis, the *ΔyopB* Y. enterocolitica mutant was used in this study. Y. enterocolitica *ΔyopB* mutant possesses T3SS, along with all six effector proteins (Yops), but is unable to translocate effector proteins into the host cytosol (46). THP-1 macrophages were exposed to equal amounts of viable Y. enterocolitica, Δ*yopB* Y. enterocolitica, or heat-treated Y. enterocolitica (multiplicity of infection [MOI] of 50) for 2 h, followed by quantification of a PGE2 and IL-1β by enzyme-linked immunosorbent assay (ELISA). PGE2 secretion was upregulated in macrophages in response to both heat-killed and Δ*yopB* Y. enterocolitica infection but not in cells infected with the wild-type bacteria (Fig. 1A). In previous study, we demonstrated that during Y. enterocolitica infection of THP-1 macrophages, there is a positive regulatory loop between PGE2 and IL-1β (13). Here, we report that IL-1β release in THP-1 macrophages is significantly increased in response to heat-killed Y. enterocolitica treatment and Δ*yopB* Y. enterocolitica infection, but not in wild-type Y. enterocolitica infection (Fig. 1B).

**FIG 2:**
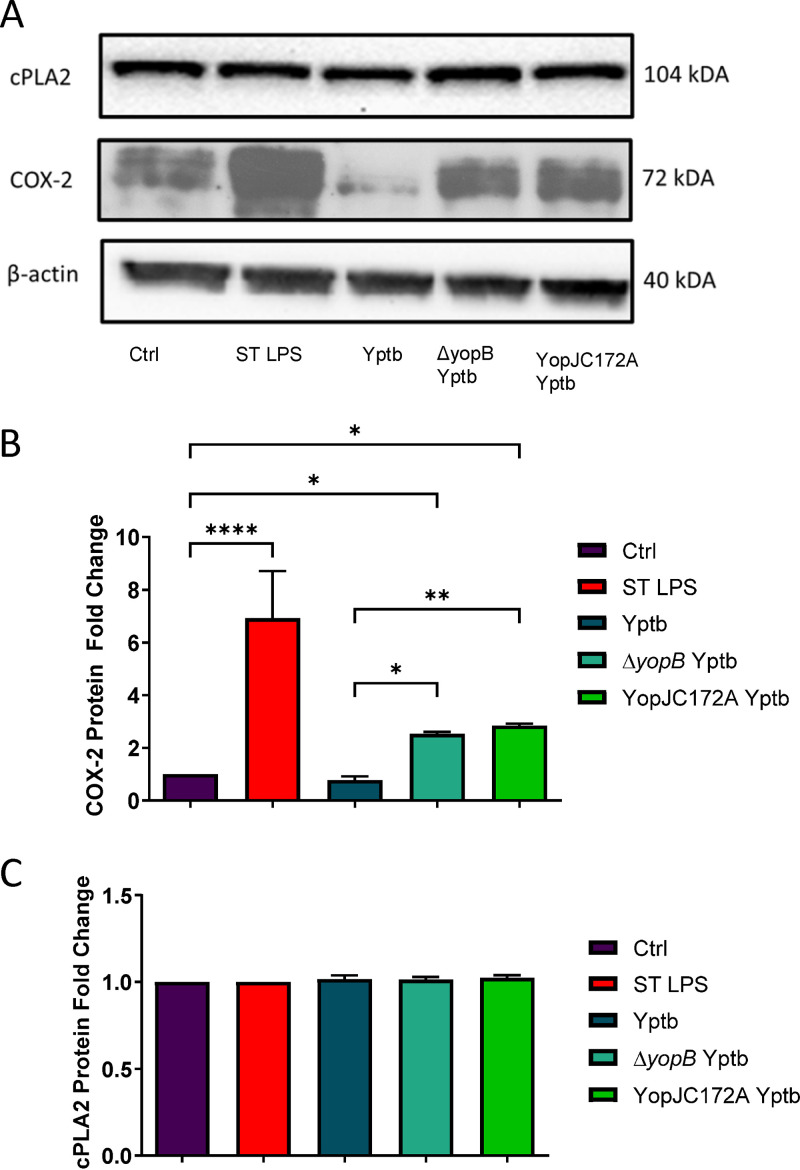
Analysis of COX-2 and cPLA2 transcript and protein expression in THP-1 macrophages after the treatment with viable wild type, heat-killed, Δ*yopB*, and YopJC172A Y. pseudotuberculosis. PMA-differentiated THP-1 macrophages were infected (or remained uninfected, Ctrl) with live or heat-killed wild type, Δ*yopB*, and YopJC172A Y. pseudotuberculosis (Yptb) for 2 h. LPS isolated from S. enterica (ST LPS) was used to treat THP-1 macrophages as a positive control. At 2 hpi, cells were collected and lysed, followed by the separation of proteins by SDS-PAGE. (A) Western blotting was performed with specific antibodies to COX-2 and cPLA2. (B and C) Protein fold change was calculated from densitometry analysis using ImageJ, where the protein levels of targets were normalized to β-actin (loading control). Unpaired Student *t* tests were used to calculate statistical significance from Western blot densitometry (*n* = 3). (D) The COX-2 mRNA fold change compared to vehicle control-treated cells was calculated using Bio-Rad CFX-manager software and normalized to GAPDH transcript levels (*n* = 3). *, *P* ≤ 0.05; **, *P* ≤ 0.01; ***, *P* ≤ 0.001; ****, *P* ≤ 0.0001. The images shown are representative of three independent experiments.

Having determined that Y. enterocolitica Δ*yopB* induces similar levels of PGE2 secretion in macrophages to the heat-killed Y. enterocolitica treatment, we next sought to identify whether Y. pseudotuberculosis exhibited a similar phenotype in infected THP-1 macrophages. This bacterium was examined because Y. pseudotuberculosis is more closely related (∼97% nucleotide similarity for over 75% of the genes) to Y. pestis, which is a significant bioterrorism threat and the causative agent of bubonic plague (47). This experiment was important to determine whether inhibition of PGE2 biosynthesis by *Yersinia* infection was conserved across multiple yersiniae. Indeed, PGE2 biosynthesis was most prominent in THP-1 macrophages treated with heat-killed and Δ*yopB* Y. pseudotuberculosis, while wild-type Y. pseudotuberculosis did not induce PGE2 biosynthesis under the tested conditions (Fig. 2C). Similarly, heat-killed Y. pseudotuberculosis and Δ*yopB* Y. pseudotuberculosis induced high levels of IL-1β secretion in THP-1 macrophages, whereas wild-type bacteria did not have this effect (Fig. 2D).

### *Y. enterocolitica* and *Y. pseudotuberculosis* require a functional T3SS to inhibit PGE2 and IL-1β secretion from THP-1 macrophages during infection.

Since translocation of bacterial effectors has been determined to be important for the *Yersinia*-mediated inhibition of PGE2, we next speculated that one of the Yops is responsible for PGE2 inhibition. We hypothesized that YopJ attenuates the host response to LPS, including inflammasome activation. YopJ is a virulence factor encoded by Y. pseudotuberculosis that inactivates host MAPK signaling pathways, including MAPK/ERK (MEK) and Jun kinases (JUN) (41, 48). We hypothesized that YopJ attenuates the host response to LPS, including inflammasome activation.

First, it was essential to show whether YopJ controls the changes in PGE2 secretion during *Yersinia* infection by regulating the expression of the key PGE2 biosynthetic enzymes COX-2 and cPLA2. Western blot analysis of the PGE2 biosynthetic enzymes COX-2 and cPLA2 was performed to determine whether the protein abundance of these enzymes was altered in macrophages in response to Y. pseudotuberculosis infection. THP-1 macrophages were infected with wild-type, heat-killed, Δ*yopB*, or YopJC172A Y. pseudotuberculosis strains for 2 h at an MOI of 50:1, followed by Western blotting of COX-2 and cPLA2 protein levels. As previously reported, the YopJC172A mutant of Y. pseudotuberculosis contains a catalytically inactive YopJ acetyltransferase (48). Live wild-type Y. pseudotuberculosis downregulated COX-2 protein levels compared to uninfected THP-1 macrophages. Moreover, COX-2 protein levels were increased in response to heat-killed, Δ*yopB*, and a YopJC172A mutant Y. pseudotuberculosis compared to uninfected control (Fig. 2A and B). Simultaneously, cPLA2 levels remained unchanged in response to Y. pseudotuberculosis (Fig. 2A and C).

### Shotgun proteomics and pathway analysis predict that *Y. pseudotuberculosis* YopJ downregulates host response to LPS by altering PGE2 biosynthesis in infected THP-1 macrophages.

**FIG 3:**
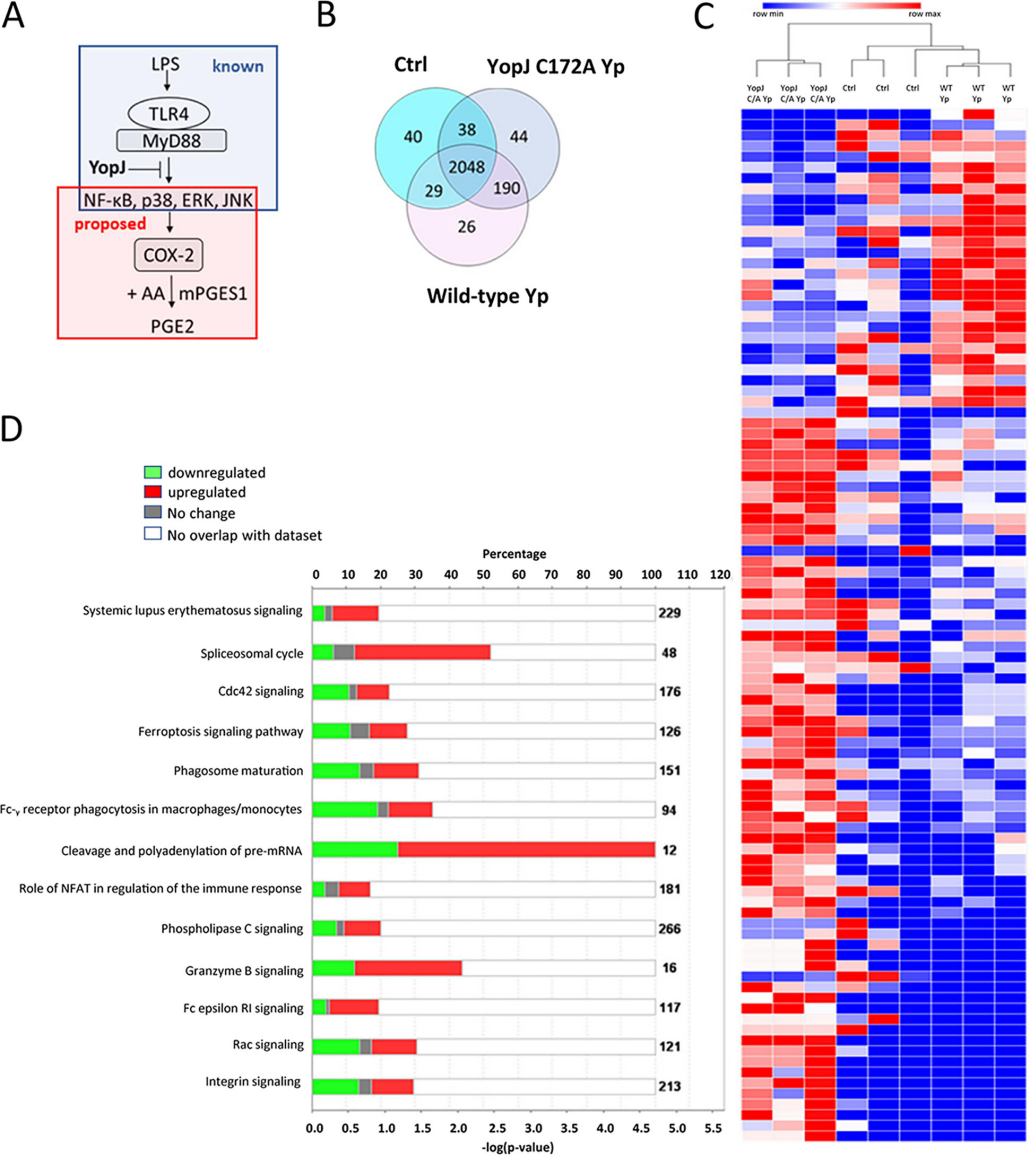
Shotgun proteomics and pathway analysis of THP-1 macrophages infected with wild-type or YopJC172A mutant Y. pseudotuberculosis. PMA-differentiated THP-1 macrophages were infected at an MOI of 50:1 with wild-type or YopJC172A Y. pseudotuberculosis for 2 h. Cell pellets were lysed and separated by SDS-PAGE, and entire lanes were subjected to tryptic digestion, followed by mass spectrometry-based analysis (*n* = 3). (A) Model of YopJ-mediated inhibition of macrophage response to bacterial LPS. (B) Venn diagram shows a summary of differential protein identifications in wild-type or YopJC172A Y. pseudotuberculosis-infected macrophages. (C) Heat map of augmented proteins in the proteomic analysis analyzed by hierarchical clustering. The grouped samples include identifications in wild-type (WT *Yp*) or YopJC172A (YopJ C/A) mutant strains of Y. pseudotuberculosis-infected macrophages, as well as uninfected cells (Ctrl). (D and E) Canonical pathway analysis of proteins differentially abundant in panel E. Y. pseudotuberculosis-infected macrophages compared to uninfected cells (D) or YopJC172A mutant of Y. pseudotuberculosis-infected macrophages in comparison to wild-type Y. pseudotuberculosis. The graphs represent downregulated proteins (green), upregulated proteins (red), and proteins with unchanged abundance (gray). (F and G) The top upstream regulator identified in YopJC172A Y. pseudotuberculosis-infected macrophages in comparison to wild-type Y. pseudotuberculosis. The graphs represent downregulated proteins (green), upregulated proteins (red), molecules predicted to be activated (orange; z-score ≥ 2). Orange and blue dashed lines with arrows indicate indirect activation and inhibition, respectively, while solid lines indicate direct effects. Lipopolysaccharide (LPS) signaling (z-score = +2.223) and (C) EGR1 (z-score = +2.162) are predicted to be activated in YopJC172A Y. pseudotuberculosis-infected macrophages compared to wild-type Y. pseudotuberculosis infection. This pathway analysis also indicated the upregulation of proteins involved in the eicosanoid biosynthetic enzymes.

Next, mass spectrometry was used to get insight into the mechanism by which YopJ inhibits PGE2 in host cells (Fig. 3A). Specifically, changes in protein abundance were compared between macrophages infected with wild-type Y. pseudotuberculosis and catalytically inactive YopJC172A Y. pseudotuberculosis. A second comparison was made between the wild-type Y. pseudotuberculosis-infected macrophages and uninfected cells. Together, 2,048 proteins were detected in all three sample types, and there were additional 367 proteins unique to one of the analyzed sample groups. In comparison to uninfected cells, YopJC172A Y. pseudotuberculosis-infected cells had unique 234 proteins. Also, compared to the Y. pseudotuberculosis-infected cells, YopJC172A mutant-infected samples had 82 unique proteins (Fig. 3B; see also Tables S1 and S2 in the supplemental material). The proteomic analysis also identified 212 *Yersinia* proteins (see Table S2).

The hierarchical clustering of differentially abundant proteins detected that uninfected cells and wild-type Y. pseudotuberculosis-infected cells were similar in terms of protein abundance compared to the YopJC172A Y. pseudotuberculosis-infected cells the latter samples clustered to a distinct group (Fig. 3C). Spectral quantification identified several critical differences between uninfected cells and wild-type Y. pseudotuberculosis*-* or YopJC172A Y. pseudotuberculosis*-*infected THP-1 macrophages (see Tables S1 and S2; see also Fig. S1). First, the canonical pathway analysis of distinct host pathways affected by wild-type Y. pseudotuberculosis infection compared to uninfected cells revealed that proteins affected by infection include such pathways as Cdc42 signaling, ferroptosis signaling pathway, phagosome maturation, Fc-gamma receptor-mediated phagocytosis in macrophages and monocytes, and other functions (Fig. 3D). Second, canonical pathway analysis of proteins affected in cells infected with YopJC172A Y. pseudotuberculosis compared to wild-type Y. pseudotuberculosis (Table 1; see also Tables S1 and S2) revealed specific pathways affected by the strain harboring YopJ catalytic mutant. These pathways were related to the sirtuin signaling pathway, NRF2-mediated oxidative stress response, oxidative phosphorylation, Rac signaling, Cdc42 signaling, mitochondrial dysfunction, and other functions (Fig. 1E). Moreover, the absence of catalytically active YopJ from cells infected with *Yersinia* upregulated mitochondrial enzymes responsible for oxidative phosphorylation, such as COX17 (Q14061, fold change [FC] = 3.5), NDUFA4 (O00483, FC = 4.1), NDUFA12 (Q9UI09, FC = 1.8), or NDUFS2 (O75306, FC = 3.7) (see Fig. S2A and B). These data suggest that YopJ might directly or indirectly affect host NAD^+^ output during infection, which should be carefully addressed in future studies. Finally, KDM5A was an upstream regulator predicted to be inhibited in YopJC172A Y. pseudotuberculosis-infected cells, leading to the upregulation of COX17, IDH3B, NDUFS2, and NDUFA4 (see Fig. S2C).

**TABLE 1:** Differentially abundant human proteins identified in YopJC172A Y. pseudotuberculosis-infected THP-1 macrophages in comparison to wild-type *Yersinia*-infected macrophages^*a*^

Symbol	Gene name	UniProt accession no.	*P*	Fold change	Location	Molecule type
DYNC1LI1	Dynein cytoplasmic 1 light intermediate chain 1	Q9Y6G9	0.021	−14.286	Cytoplasm	Other
PUS1	Pseudouridine synthase 1	Q9Y606	0.021	−14.286	Nucleus	Enzyme
UFD1	Ubiquitin recognition factor in ER associated degradation 1	Q92890	0.034	−12.5	Cytoplasm	Peptidase
EEF1E1	Eukaryotic translation elongation factor 1 epsilon 1	O43324	0.004	−3.333	Cytoplasm	Translation regulator
ACADS	Acyl-CoA dehydrogenase short chain	P16219	0.021	−2.5	Cytoplasm	Enzyme
CAPRIN1	Cell cycle-associated protein 1	Q14444	0.013	−2.5	Plasma membrane	Other
H1-10	H1.10 linker histone	Q92522	0.043	−2.5	Nucleus	Other
LUC7L3	LUC7 like 3 pre-mRNA splicing factor	O95232	0.042	−2.5	Nucleus	Other
USP14	Ubiquitin-specific peptidase 14	P54578	0.002	−2.5	Cytoplasm	Peptidase
APBB1IP	Amyloid beta precursor protein binding family B member 1 interacting protein	Q7Z5R6	0.046	−2	Cytoplasm	Other
ARF1	ADP ribosylation factor 1	P84077	0.0041	−2	Cytoplasm	enzyme
FCGR1A	Fc fragment of IgG receptor Ia	P12314	0.047	−2	Plasma membrane	Transmembrane receptor
HIGD1A	HIG1 hypoxia-inducible domain family member 1A	Q9Y241	0.0076	−2	Cytoplasm	Other
LAMTOR1	Late endosomal/lysosomal adaptor, MAPK, and MTOR activator 1	Q6IAA8	0.044	−2	Plasma membrane	Other
MEPCE	Methylphosphate capping enzyme	Q7L2J0	0.039	−2	Other	Enzyme
TAOK3	TAO kinase 3	Q9H2K8	0.019	−2	Cytoplasm	Kinase
TERF2IP	TERF2 interacting protein	Q9NYB0	0.045	−2	Nucleus	Other
TIA1	TIA1 cytotoxic granule associated RNA binding protein	P31483	0.0059	−2	Nucleus	Other
TMX1	Thioredoxin-related transmembrane protein 1	Q9H3N1	0.017	−2	Cytoplasm	Enzyme
LGALS9	Galectin 9	O00182	0.006	−1.667	Plasma membrane	Other
MAPK1	Mitogen-activated protein kinase 1	P28482	0.031	−1.667	Cytoplasm	Kinase
PSAT1	Phosphoserine aminotransferase 1	Q9Y617	0.026	−1.667	Cytoplasm	Enzyme
SCAMP3	Secretory carrier membrane protein 3	O14828	0.0018	−1.667	Cytoplasm	Transporter
SIRPA	Signal regulatory protein alpha	P78324	0.015	−1.667	Plasma membrane	Phosphatase
SNX9	Sorting nexin 9	Q9Y5X1	0.0054	−1.667	Cytoplasm	Transporter
TARS1	Threonyl-tRNA synthetase 1	P26639	0.011	−1.667	Nucleus	Enzyme
TSN	Translin	Q15631	0.03	−1.667	Nucleus	Other
ARPC2	Actin-related protein 2/3 complex subunit 2	O15144	0.049	1.5	Cytoplasm	Other
FTL	Ferritin light chain	P02792	0.0081	1.5	Cytoplasm	Enzyme
THRAP3	Thyroid hormone receptor associated protein 3	Q9Y2W1	0.045	1.5	Nucleus	Transcription regulator
LMNB2	Lamin B2	Q03252	0.0029	1.6	Nucleus	Other
NUP155	Nucleoporin 155	O75694	0.034	1.6	Nucleus	Other
SCLY	Selenocysteine lyase	Q96I15	0.0001	1.6	Cytoplasm	Enzyme
HNRNPUL2	Heterogeneous nuclear ribonucleoprotein U like 2	Q1KMD3	0.029	1.7	Nucleus	Other
EIF3I	Eukaryotic translation initiation factor 3 subunit I	Q13347	0.026	1.8	Cytoplasm	Translation regulator
GATM	Glycine amidinotransferase	P50440	0.038	1.8	Cytoplasm	Enzyme
HNRNPL	Heterogeneous nuclear ribonucleoprotein L	P14866	0.033	1.8	Nucleus	Other
NDUFA12	NADH:ubiquinone oxidoreductase subunit A12	Q9UI09	0.042	1.8	Cytoplasm	Enzyme
PCYOX1L	Prenylcysteine oxidase 1 like	Q8NBM8	0.026	1.8	Other	Other
RPL17	Ribosomal protein L17	P18621	0.035	1.8	Cytoplasm	Other
TARS2	Threonyl-tRNA synthetase 2, mitochondrial	Q9BW92	0.026	1.8	Cytoplasm	Enzyme
C1QBP	Complement C1q binding protein	Q07021	0.032	1.9	Cytoplasm	Transcription regulator
DNAJB11	DnaJ heat shock protein family (Hsp40) member B11	Q9UBS4	0.031	1.9	Cytoplasm	Other
H6PD	Hexose-6-phosphate dehydrogenase/glucose 1-dehydrogenase	O95479	0.026	1.9	Cytoplasm	Enzyme
PLOD1	Procollagen-lysine,2-oxoglutarate 5-dioxygenase 1	Q02809	0.037	2.1	Cytoplasm	Enzyme
MOGS	Mannosyl-oligosaccharide glucosidase	Q13724	0.029	2.2	Cytoplasm	Enzyme
NUP205	Nucleoporin 205	Q92621	0.021	2.2	Nucleus	Other
ITGA4	Integrin subunit alpha 4	P13612	0.036	2.3	Plasma membrane	Transmembrane receptor
NUP214	Nucleoporin 214	P35658	0.038	2.3	Nucleus	Transporter
PCYT1A	Phosphate cytidylyltransferase 1, choline, alpha	P49585	0.038	2.3	Cytoplasm	Enzyme
PREP	Prolyl endopeptidase	P48147	0.025	2.3	Cytoplasm	Peptidase
RANBP3	RAN binding protein 3	Q9H6Z4	0.04	2.4	Nucleus	Other
CLTA	Clathrin light chain A	P09496	0.049	2.5	Plasma membrane	Other
AGO2	Argonaute RISC catalytic component 2	Q9UKV8	0.044	2.7	Cytoplasm	Translation regulator
MRPS7	Mitochondrial ribosomal protein S7	Q9Y2R9	0.044	2.7	Cytoplasm	Other
PDS5A	PDS5 cohesin associated factor A	Q29RF7	0.021	2.7	Nucleus	Other
ALOX5AP	Arachidonate 5-lipoxygenase activating protein	P20292	0.0044	2.8	Plasma membrane	Other
BCLAF1	BCL2 associated transcription factor 1	Q9NYF8	0.048	3	Nucleus	Transcription regulator
HDHD2	Haloacid dehalogenase like hydrolase domain containing 2	Q9H0R4	0.0016	3	Cytoplasm	Other
CALU	Calumenin	O43852	0.024	3.1	Cytoplasm	Other
CBX3	Chromobox 3	Q13185	0.022	3.1	Nucleus	Transcription regulator
ZC3H4	Zinc finger CCCH-type containing 4	Q9UPT8	0.048	3.1	Nucleus	Transcription regulator
TM9SF4	Transmembrane 9 superfamily member 4	Q92544	0.0087	3.3	Cytoplasm	Transporter
COX17	Cytochrome *c* oxidase copper chaperone COX17	Q14061	0.036	3.5	Cytoplasm	Enzyme
NDUFS2	NADH:ubiquinone oxidoreductase core subunit S2	O75306	0.04	3.7	Cytoplasm	Enzyme
NDUFA4	NDUFA4 mitochondrial complex associated	O00483	0.016	4.1	Cytoplasm	Enzyme
PPIF	Peptidylprolyl isomerase F	P30405	0.0026	4.1	Cytoplasm	Enzyme
GGH	Gamma-glutamyl hydrolase	Q92820	0.034	4.4	Cytoplasm	Peptidase
RALY	RALY heterogeneous nuclear ribonucleoprotein	Q9UKM9	0.045	4.8	Nucleus	Transcription regulator
TSFM	Ts translation elongation factor, mitochondrial	P43897	0.022	4.8	Cytoplasm	Translation regulator
MRPL45	Mitochondrial ribosomal protein L45	Q9BRJ2	0.021	4.9	Cytoplasm	Other
PPP5C	Protein phosphatase 5 catalytic subunit	P53041	0.021	4.9	Nucleus	Phosphatase
TRA2B	Transformer 2 beta homolog	P62995	0.02	6.1	Nucleus	Other
GUK1	Guanylate kinase 1	Q16774	0.0001	8.8	Cytoplasm	Kinase
ATAD3A	ATPase family AAA domain containing 3A	Q9NVI7	0.019	12	Cytoplasm	Other
EDF1	Endothelial differentiation related factor 1	O60869	0.019	12	Nucleus	Transcription regulator
GOLGB1	Golgin B1	Q14789	0.019	12	Cytoplasm	Other
IDH3B	Isocitrate dehydrogenase (NAD^+^) 3 noncatalytic subunit beta	O43837	0.019	12	Cytoplasm	Enzyme
RPS6KA3	Ribosomal protein S6 kinase A3	P51812	0.025	14	Cytoplasm	Kinase
GET3	Guided entry of tail-anchored proteins factor 3, ATPase	O43681	0.0001	18	Nucleus	Transporter
MRPL39	Mitochondrial ribosomal protein L39	Q9NYK5	0.0001	18	Cytoplasm	Other
PFDN4	Prefoldin subunit 4	Q9NQP4	0.0001	18	Cytoplasm	Other
HAX1	HCLS1-associated protein X-1	O00165	0.0021	21	Cytoplasm	Other
RSL1D1	Ribosomal L1 domain containing 1	O76021	0.028	21	Nucleus	Other
SQLE	Squalene epoxidase	Q14534	0.0021	21	Cytoplasm	Enzyme
JUN	Jun proto-oncogene, AP-1 transcription factor subunit	P05412	0.0014	23	Nucleus	Transcription regulator
RNASE2	RNase A family member 2	P10153	0.0066	26	Cytoplasm	Enzyme
NDRG1	N-myc downstream regulated 1	Q92597	0.02	29	Nucleus	Kinase
TOMM40	Translocase of outer mitochondrial membrane 40	O96008	0.016	35	Cytoplasm	Ion channel

a The table includes a gene designation, the Entrez gene name, the UniProt accession number, the experimental *P* value calculated using a Student *t* test, the experimental fold change value calculated based on the normalized and weighted spectral count (YopJC172A *Yersinia*:wild-type *Yersinia*), the protein location, and the molecule type.

Upstream regulator analysis indicated that the putative host LPS response was upregulated in YopJC172A Y. pseudotuberculosis-infected cells in comparison to wild-type Y. pseudotuberculosis infection (Fig. 3F), including predicted upregulation of COX-2 (PTGS2) and PGE2 (Fig. 3F). Interestingly JUN (c-JUN; AP-1, P05412) was highly upregulated in response to YopJC172A Y. pseudotuberculosis in comparison to wild-type infection (fold change, FC = 23), indicating a potential role for this transcriptional factor in mediating the host response to LPS and specific effects on PGE2 (Fig. 3F; see also Table S1). In addition, upregulation of the transcription factor early growth response (EGR1) in the YopJC172A Y. pseudotuberculosis-infected macrophages was predicted. LPS has been previously shown to activate EGR transcription in peritoneal macrophages (49, 50), and combined with our data, EGR might mediate LPS-mediated activation of proinflammatory gene transcription in response to ERK1/2 activation by *Yersinia* LPS. Collectively, our bioinformatic analysis predicted that the YopJ effector suppresses host responses to bacterial LPS, including downregulation of PGE2 biosynthesis via COX-2.

### YopJ effector or chemical inhibition of MAPK/ERK signaling diminishes PGE2 biosynthesis in response to *Y. pseudotuberculosis* infection of THP-1 macrophages.

**FIG 4:**
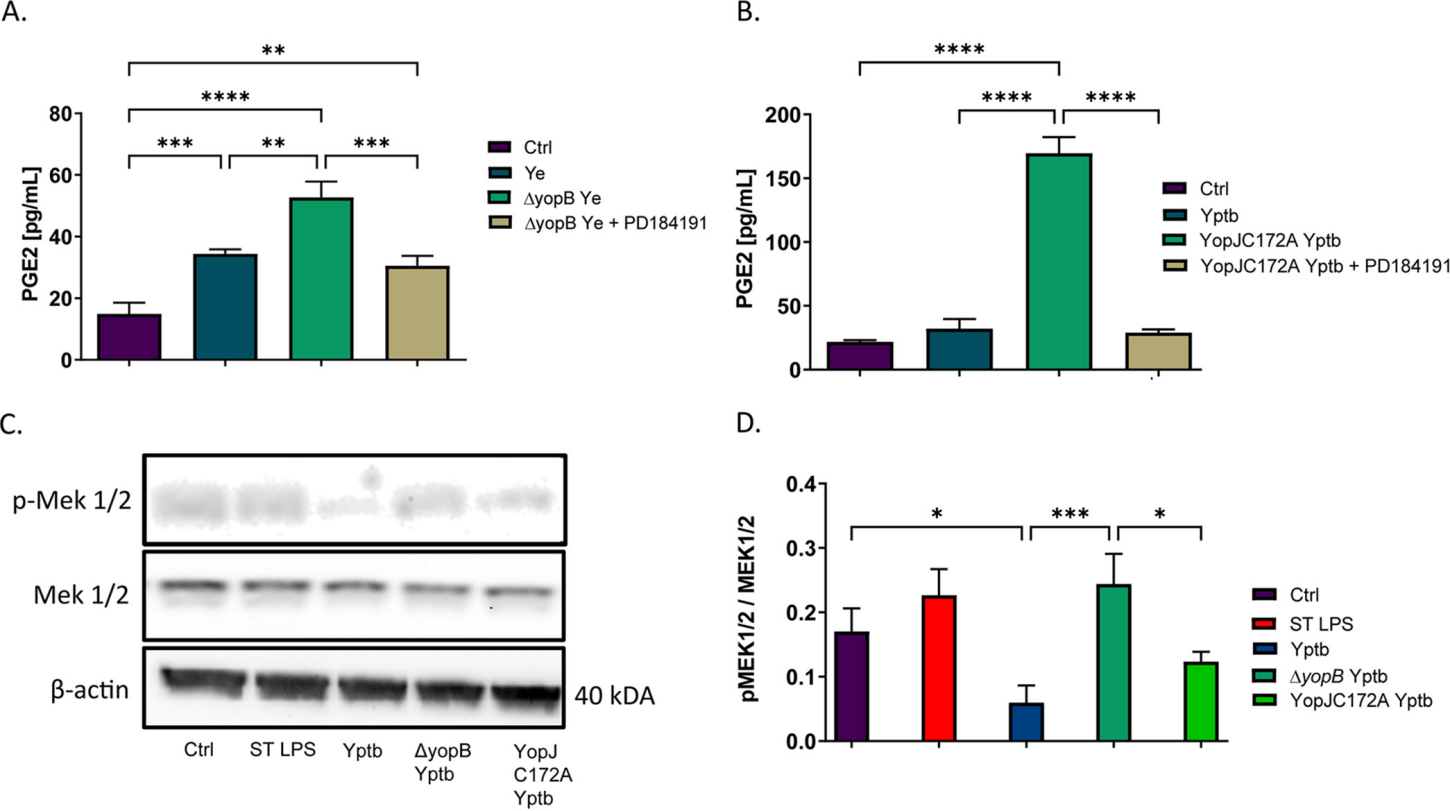
Analysis of PGE2 biosynthesis and MEK function in THP-1 macrophages in response to treatment with *ΔyopB* Y. enterocolitica, YopJC172A Y. pseudotuberculosis, or the chemical MEK inhibitor PD184161. PMA-differentiated THP-1 macrophages were pretreated with or without 10 μM PD184161 (MEK1/2 inhibitor) 1 h before infection. Macrophages were uninfected (Ctrl) or were infected with wild-type or *ΔyopB* Y. enterocolitica (Ye) (A) or wild-type or YopJC172A Y. pseudotuberculosis (Yptb) (B and C) for 2 h at an MOI of 50:1. The PGE2 concentration from infected cell supernatants was measured by a commercial monoclonal ELISA recognizing PGE2 (A and B). Western blotting was used to quantify MEK1/2 and phosphorylated MEK/2 (C and D), where the fold change was calculated using densitometry analysis by normalizing to phosphorylated MEK 1/2 to MEK1/2 levels (D). The β-actin was used as a loading control. Two-way ANOVA and Tukey’s *post hoc* test were used to calculate significance for Western blot densitometry (*n* = 3). *P* values are indicated (*, *P* ≤ 0.05; **, *P* ≤ 0.01; ***, *P* ≤ 0.001; ****, *P* ≤ 0.0001). The images shown are representative of three independent experiments.

We subsequently investigated the mechanism behind COX-2 induction during *Yersinia* infection involving specific cellular signaling pathways predicted by proteomics studies. COX-2 transcription can be regulated by MAPKs. The MEK pathway is one of the pathways that induce proinflammatory cytokine release in response to various stimuli, including TLR stimulation by LPS (7, 21, 51). MEK kinase is rapidly phosphorylated in response to Y. pseudotuberculosis infection, but MEK is dephosphorylated over time in the presence of YopJ (48). We hypothesized that MEK activation is involved in COX-2 induction during *ΔyopB* Y. enterocolitica or YopJC172A Y. pseudotuberculosis infection. The chemical antagonist of the MEK pathway, PD184161, was used to block TLR signal transduction during infection of THP-1 macrophages with either *ΔyopB* Y. enterocolitica or YopJC172A Y. pseudotuberculosis. As a negative control, THP-1 macrophages were infected with wild-type Y. enterocolitica or Y. pseudotuberculosis since these strains limit PGE2 production (Fig. 4A and B) or left uninfected. As a positive control, *S.* Typhimurium LPS treatment was used. We found that PGE2 biosynthesis was elevated in THP-1-derived macrophages infected with either *ΔyopB* Y. enterocolitica or YopJC172A Y. pseudotuberculosis, but PGE2 was diminished when the cells were treated with the MEK inhibitor PD184161 (Fig. 4A and B). MEK activity during wild-type and YopJC172A Y. pseudotuberculosis infection was evaluated by Western blotting with antibodies against phospho-MEK at Ser 217/221 residue (Fig. 4C). Ser 217/221 residue is the target of YopJ transacetylation involved in the inhibition of autophosphorylation of downstream kinases (43). Phosphorylation of MEK at Ser 217/221 was increased in Δ*yopB* Y. pseudotuberculosis*-*infected cells, as well as YopJC172A Y. pseudotuberculosis-infected cells. MEK phosphorylation was inhibited in wild-type Y. pseudotuberculosis*-*infected THP-1 macrophages compared to uninfected macrophages or YopJC172A Y. pseudotuberculosis*-*infected macrophages (Fig. 4D).

### Y. pseudotuberculosis inhibits pro-IL-1β mRNA transcription and expression in response to wild-type but not *ΔyopB* or YopJC172A Y. pseudotuberculosis*-*infected THP-1 macrophages.

EP4 signaling was previously shown to be engaged in caspase-1-dependent IL-1β secretion during Y. enterocolitica infection (13). PGE2 binding to EP2/EP4 receptors stimulates activation of adenylate cyclase, leading to a cAMP increase and downstream NF-κB activation (52–54). Alternatively, PGE2 binding to EP4 receptor can also activate the phosphoinositide 3-kinase signaling cascade (55, 56). In response to LPS binding to TLR4 and signal transduction via MAPKs, pro-IL-1β is synthesized, which can be regulated by the availability of endogenous PGE2 (17, 51, 57, 58). Upon inflammasome activation, pro-IL-1β is cleaved by active caspase-1 to mature IL-1β, followed by secretion of this cytokine. IL-1β acts locally on cells by binding to IL-1R on cell surface membranes and inducing inflammation (57, 59).

**FIG 5:**
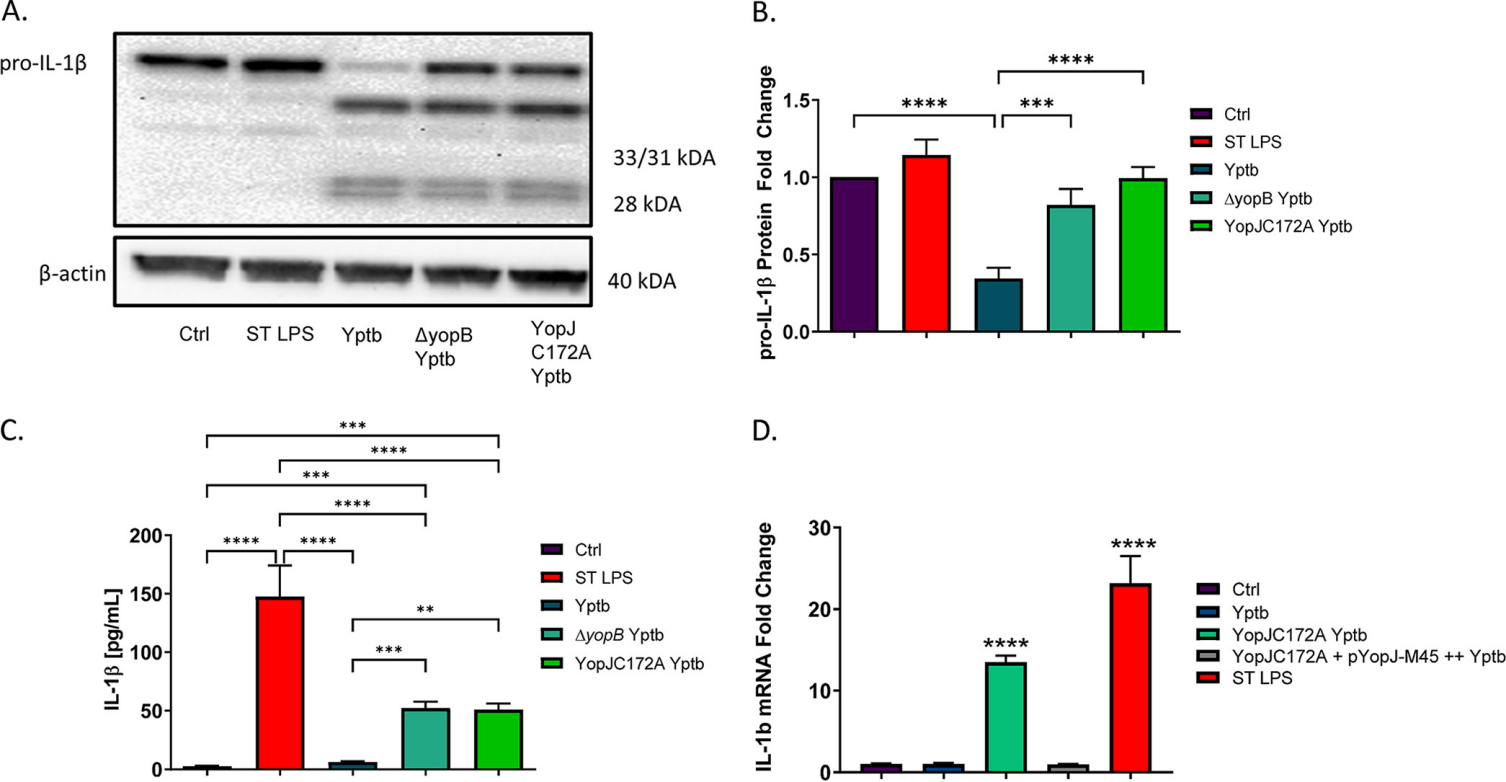
Expression of pro-IL-1β from THP-1 macrophages infected with YopJC172A Y. pseudotuberculosis. PMA-differentiated THP-1 macrophages were infected with wild-type, *ΔyopB*, or YopJC172A mutant Y. pseudotuberculosis (Yptb) at an MOI of 50:1 for 2 h or left uninfected (Ctrl). As a positive control, 10 μg/ml S. enterica LPS was used to treat cells instead for 2 h after PMA differentiation. Cell pellets were collected for Western blotting (A) and qRT-PCR (B and D) analyses. Protein fold changes in IL-1β were calculated using densitometry analysis and were normalized to β-actin (loading control). (C) The IL-1β concentration was measured in cell supernatants at 2 hpi using ELISA. Two-way ANOVA and Tukey’s *post hoc* test was used to calculate significance for Western blot analysis (*n* = 3). (D) THP-1 macrophages were infected with wild-type Y. pseudotuberculosis (Yptb), YopJC172A, or YopJC172A mutant expressing YopJ (YopJ-M45) at an MOI of 50:1 for 2 h. YopJC172A+pYopJ-M45 strain was grown in the presence of 0.1 mM IPTG before infection. Alternatively, cells were treated with LPS from Salmonella (ST LPS). The qRT-PCR analysis was used to analyze pro-IL-1β transcripts, where pro-IL-1β mRNA fold change was calculated by normalizing it to GAPDH transcript levels and using a *t* test to indicate significance (Bio-Rad CFX-manager software; *n* = 3). *P* values are indicated (*, *P* ≤ 0.05; **, *P* ≤ 0.01; ***, *P* ≤ 0.001; ****, *P* ≤ 0.0001). The data are representative of three independent experiments.

In the present study, YopJ was tested as an effector that contributed to the inhibition of PGE2 biosynthesis in infected THP-1 macrophages upstream to IL-1β synthesis. THP-1 macrophages were infected with wild-type, Δ*yopB*, or YopJC172A Y. pseudotuberculosis or treated with S. enterica LPS (positive control), followed by RT-PCR and Western blot analysis of pro-IL-1β. Wild-type Y. pseudotuberculosis downregulated pro-IL-1β synthesis compared to treatment with S. enterica LPS and Δ*yopB* or YopJC172A Y. pseudotuberculosis (Fig. 5A and B). Secreted IL-1β was upregulated in response to S. enterica LPS, Δ*yopB*, or YopJC172A Y. pseudotuberculosis-infected macrophages but not wild-type or uninfected cells (Fig. 5C). Moreover, *il-1b* mRNA transcripts were significantly upregulated in response to S. enterica LPS and YopJC172A Y. pseudotuberculosis but not to wild-type Y. pseudotuberculosis infection (Fig. 5D). These results confirm that IL-1β secretion and expression in THP-1 macrophages depend on the presence of catalytically active YopJ.

### Ectopic expression of YopJ in the catalytically inactive YopJC172A mutant of Y. pseudotuberculosis reduces PGE2 biosynthesis in infected THP-1 macrophages by downregulating COX-2 mRNA.

**FIG 6:**
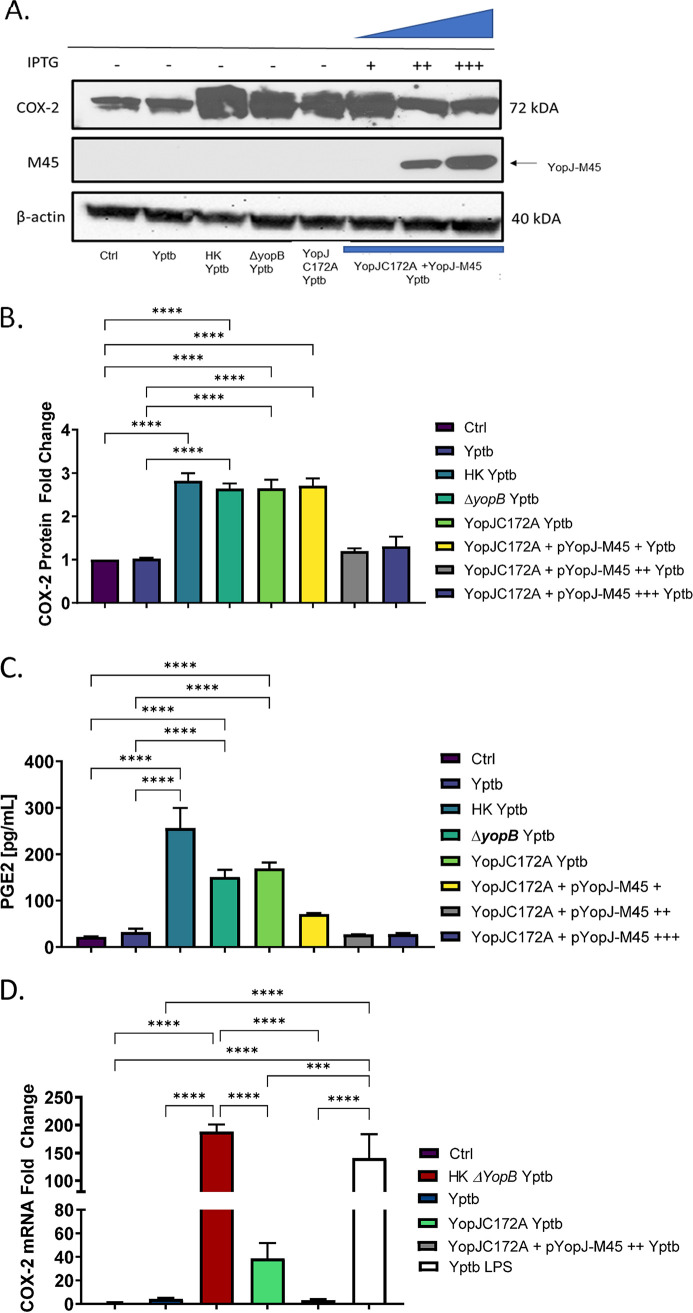
Effect of ectopic expression of YopJ on host COX-2 and PGE2 biosynthesis in Y. pseudotuberculosis*-*infected THP-1 macrophages. (A to C) PMA-differentiated THP-1 macrophages were treated with heat-killed Y. pseudotuberculosis or infected with live wild-type Y. pseudotuberculosis, Δ*yopB*, YopJC172A, or YopJC172A mutant expressing YopJ (YopJ-M45) at an MOI of 50:1 for 2 h. The YopJC172A-pYopJ-M45 strain contains a plasmid with the wild-type YopJ sequence under the control of an IPTG-inducible promoter. YopJC172A+pYopJ-M45 strain was grown in the presence of 0.01, 0.1, and 0.4 mM IPTG before infection of THP-1 macrophages. The protein fold change was calculated from densitometry analysis (ImageJ) and normalized to β-actin (loading control). Two-way ANOVA and Tukey’s *post hoc* test were used to calculate significance from Western blot densitometry (*n* = 3). *P* values are indicated (*, *P* ≤ 0.05; **, *P* ≤ 0.01; ***, *P* ≤ 0.001; ****, *P* ≤ 0.0001). (C) PGE2 concentration in cell culture supernatants was measured using monoclonal ELISA. (D) THP-1 cells were treated with 10 μg/ml LPS isolated from Y. pseudotuberculosis grown at 37°C in LB without aeration, a gift kindly provided by Robert Ernst (60). Alternatively, cells were infected with live wild-type Y. pseudotuberculosis, heat-killed Δ*yopB*, YopJC172A Y. pseudotuberculosis, or the YopJC172A Y. pseudotuberculosis mutant expressing YopJ-M45 at an MOI of 50:1 for 2 h similarly, as described above. The RNA from cells was collected and used to measure mRNA from COX-2 and GAPDH (housekeeping control). The transcripts of COX-2 were analyzed by qRT-PCR, where COX-2 mRNA fold change was normalized to GAPDH mRNA levels. *, *P* ≤ 0.05; **, *P* ≤ 0.01; ***, *P* ≤ 0.001; ****, *P* ≤ 0.0001. The data are representative of three independent experiments.

Next, the mutant strain containing catalytically inactive YopJ (YopJC172A) was complemented with a wild-type YopJ. Here, YopJC172A Y. pseudotuberculosis was transformed with a pMMB67HE plasmid encoding a copy of the wild-type YopJ under the control of a tac promoter with a carboxy-terminal M-45 tag via conjugation with E. coli (strain is denoted as YopJC172A+pYopJ-M45). A C-terminal M45 tag attached to the YopJ protein allows for detecting recombinant YopJ in cell lysates via Western blotting with an antibody specific to the M45 tag (48). PMA differentiated THP-1 macrophages were infected with live or heat-killed wild-type, *ΔyopB*, YopJC172A mutant, and YopJC172A-pYopJ-M45 expressing wild-type YopJ using various levels of IPTG induction. Cell lysates at 2 h postinfection (hpi) were probed for COX-2 protein and mRNA levels by Western blotting and RT-PCR, respectively. Heat-killed Y. pseudotuberculosis induced the most notable increase in COX-2 protein expression, compared to uninfected or live wild-type Y. pseudotuberculosis (Fig. 6A and B). At 0.01 mM IPTG induction, there was no difference in COX-2 expression between YopJC172A and the YopJC172A strain complemented with YopJ. However, at 0.1 and 0.4 mM IPTG, COX-2 protein levels (Fig. 6A and B) and PGE2 levels (Fig. 6C) are downregulated in cells infected with YopJC172A strain complemented with YopJ compared to cells infected with wild-type *Yersinia* or uninfected control. Western blotting confirmed that YopJ (YopJ-M45) was present in the infected host cells when IPTG concentrations of 0.1 and 0.4 mM were used to induce YopJ in the infected THP-1 macrophages (Fig. 1A).

Next, RT-PCR analysis confirmed that the inhibition of COX-2 expression by Y. pseudotuberculosis YopJ was due to transcription changes. COX-2 transcripts were evaluated in THP-1 macrophages previously infected with wild-type, heat-killed Δ*yopB* (wild-type heat-killed yersiniae were tested earlier, see Fig. 1), YopJC172A mutant, and YopJC172A mutant complemented with YopJ (pYopJ-M45) Y. pseudotuberculosis. As a positive control, THP-1 macrophages were treated with LPS isolated from Y. pseudotuberculosis grown at 37°C (60), mimicking the LPS state in the infected host. The *Yersinia* LPS, after its growth at 37°C, has a different acylation state compared to *Yersinia* grown at lower temperatures. While at 37°C, Y. pseudotuberculosis contains a heterogeneous population of lipid A, which includes the tetra-acylated lipid IVA and other lipid species, but when grown at 21°C, Y. pseudotuberculosis LPS contains hexa-acylated lipid A (60) predominantly. These different acylation states affect the proinflammatory activity of this molecule (61). The COX-2 transcripts were upregulated in all treatments except wild-type Y. pseudotuberculosis and YopJC172A complemented with YopJ. The overexpression of YopJ in YopJC172A strain with dysfunctional YopJ led to the same COX-2 levels as the wild-type strain or control (uninfected) cells (Fig. 6A and B). Similarly, PGE2 was upregulated in cells exposed to heat-inactivated Y. pseudotuberculosis and to live YopJC172A Y. pseudotuberculosis. In contrast, PGE2 was not increased in cells infected with a live strain of this bacterium or cells exposed to YopJC172A Y. pseudotuberculosis complemented with YopJ (Fig. 6C and Fig. 1; see also Fig. S3). Also, COX-2 transcripts were upregulated in cells exposed to heat-inactivated Δ*yopB* Y. pseudotuberculosis or live YopJC172A, but not wild-type Y. pseudotuberculosis or YopJC172A complemented with YopJ. Moreover, cells exposed to Y. pseudotuberculosis LPS alone showed upregulation of COX-2 transcripts as well (Fig. 6D). These results indicated that LPS from this bacterium is sufficient to upregulated COX-2 transcription, but YopJ effector’s presence decreased this mRNA.

### YopJ diminishes PGE2 and IL-1β biosynthesis in response to *Y. pseudotuberculosis* infection of LPS-primed bone-marrow-derived macrophages.

**FIG 7:**
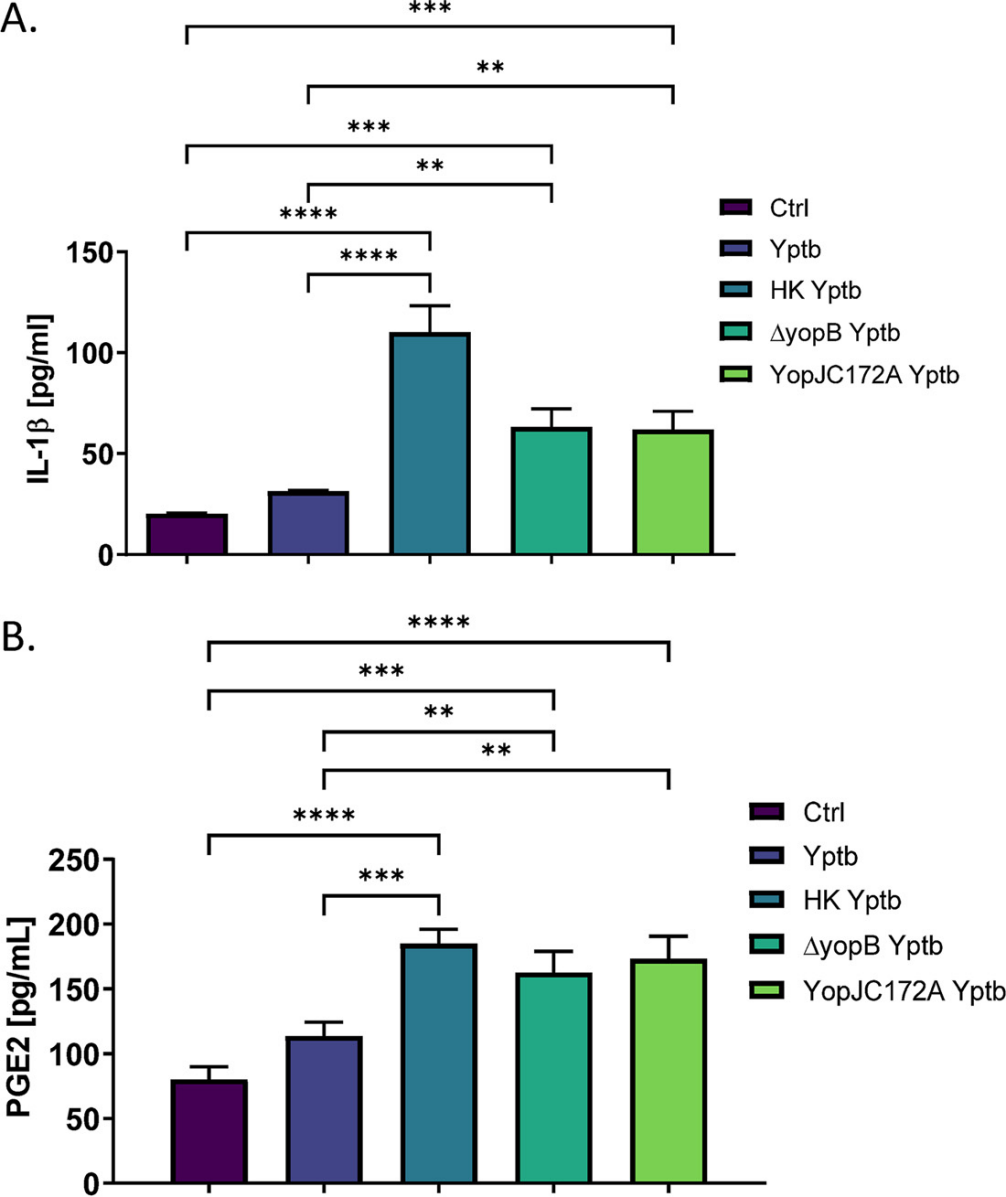
PGE2 and IL-1β secretion from LPS primed primary bone marrow-derived macrophages in response to live or heat-killed wild type, *ΔyopB*, or YopJC172A Y. pseudotuberculosis infection. Primary BMDMs isolated from wild-type BALB/c mice were primed with 10 μg/ml LPS for 1 h before infection with live or heat-killed wild-type, *ΔyopB*, or YopJC172A Y. pseudotuberculosis at an MOI of 50:1. At 2 hpi, the supernatant was collected and probed for PGE2 (A) and IL-1β (B) concentrations by commercial ELISA. Student *t* tests were used to calculate significance (*n* = 3). *P* values are indicated (*, *P* ≤ 0.05; **, *P* ≤ 0.01; ***, *P* ≤ 0.001; ****, *P* ≤ 0.0001).

Since YopJ is responsible for inhibiting PGE2 biosynthesis in a catalytically dependent manner in THP-1 macrophages, these results were further validated in primary bone marrow-derived macrophages (BMDMs) to ensure that the process is not cell specific. BMDMs were subjected to LPS stimulation for 1 h before infection with live or heat-killed wild-type, Δ*yopB* Y. pseudotuberculosis, or YopJC172A Y. pseudotuberculosis at an MOI of 50:1 for 2 h. This specific protocol was followed to induce inflammasome as previously described (48). PGE2 and IL-1β quantification in cell culture supernatant revealed that heat-killed, Δ*yopB*, or YopJC172 Y. pseudotuberculosis induced PGE2 and IL-1β secretion from infected BMDMs compared to wild-type Y. pseudotuberculosis*-*infected cells (Fig. 7).

### PGE2 signaling via EP4 affects intracellular survival of *Yersinia* in macrophages.

**FIG 8:**
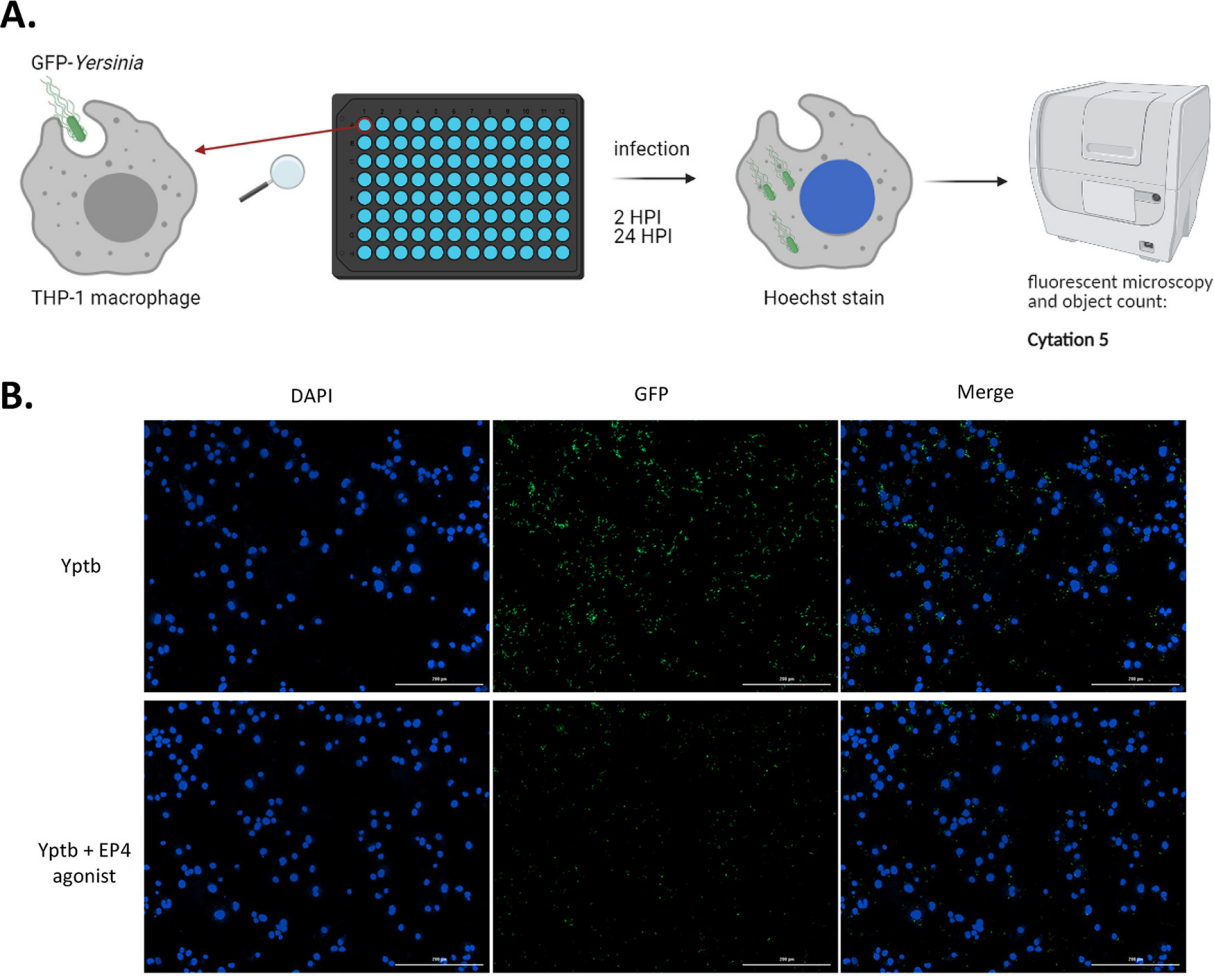
Survival of intracellular Y. pseudotuberculosis in cells treated with EP4 agonist. (A and B) THP-1 macrophages were treated with EP4 agonist L-902688, JUN inhibitor, or vehicle control (ethanol, 0.01% [vol/vol] DMSO) in RPMI lacking antibiotics for 30 min before infection with wild-type and YopJC172A GFP-Y. pseudotuberculosis (MOI of 15:1). At 1 hpi, media were removed, and cells were washed with PBS to remove extracellular bacteria. RPMI media lacking antibiotics supplemented with gentamicin (100 μg/ml), with or without EP4 agonist and with or without JUN inhibitor, were added, and the cells were incubated further for another hour. At 2 hpi, the media were removed again, and the cells were washed with PBS and resuspended in media containing a lower concentration of gentamicin (10 μg/ml) for the remainder of the infection (total, 2 and 24 hpi). For fluorescence imaging, the cells were stained with Hoechst stain, and the entire well was imaged using Cytation 5 at ×10 magnification. (C) The number of GFP-*Yersinia* per 1,000 cells was calculated by dividing total GFP cell counts by total host cell counts at 2 and 24 hpi multiplied by 1,000. (D) THP-1 cell count as determined by Hoechst staining and cell counting using GenV software. All data were analyzed by two-way ANOVA, followed by multiple testing correction (Tukey’s; *n* = 4). *P* values are indicated (*, *P* ≤ 0.05; **, *P* ≤ 0.01; ***, *P* ≤ 0.001; ****, *P* ≤ 0.0001).

Finally, the importance of PGE2 signaling in the YopJ-mediated increased intracellular survival of Y. pseudotuberculosis was tested. To achieve this goal, we designed an assay using fluorescently tagged *Yersinia* for infections (Fig. 8A). THP-1 macrophages were treated with EP4 agonist L-902688 to stimulate EP4 receptor, which was likely crucial in the PGE2-mediated effects during this infection (13). Alternatively, cells were treated with JUN inhibitor since YopJ is predicted to inhibit JUN expression (Fig. 3). Wild-type and YopJ-deficient mutant of Y. pseudotuberculosis expressing YopJC172A were used to infect cells seeded on 96-well optical black plates. The cells were previously pretreated with EP4 agonist, or JUN inhibitor, followed by infection and bacteria count at 2 and 24 hpi. Both wild-type and YopJ-deficient mutant of Y. pseudotuberculosis invaded and survived inside infected THP-1 macrophages, although the mutant of *Yersinia* devoid of YopJ has markedly less survival at 24 hpi (Fig. 8B and C). EP4 agonist did not affect phagocytosis of wild-type *Yersinia* at 2 hpi, but it contributed to a significantly decreased bacterial survival at 24 hpi (Fig. 8B and C). There was no difference in the survival of bacteria within THP-1 infected with YopJC172A Y. pseudotuberculosis treated with EP4 agonist at 2 and 24 hpi in comparison to vehicle control, likely since COX-2 and PGE2 are readily produced in cells infected with this strain, thus activating PGE2 receptor, EP4 (Fig. 6). Notably, there was no noticeable difference in the cell survival when the EP4 agonist was used (Fig. 8D). These results suggest that the PGE2 signaling via the EP4 receptor could lead to the clearance of bacteria in macrophages, a process that YopJ can partially block.

## DISCUSSION

Inflammasomes are multimolecular complexes that can sense pathogen presence and trigger a robust proinflammatory response. One of the inflammasome components, caspase-1, cleaves immature pro-IL-1β and converts it to mature IL-1β. IL-1β is then released through pores, and the pore formation can also result in inflammatory cell death, pyroptosis. The inflammasome-mediated IL-1β secretion typically serves as a host protective measure against bacteria, although this might depend on the specific pathogen. Several *Yersinia* effectors, such as YopE and YopT, can induce pyrin inflammasome (62). In contrast, YopM (40, 63) or YopJ (41) inhibit inflammasome in Y. pestis and Y. pseudotuberculosis infection. The mechanisms by which YopJ contributes to the inhibition of IL-1β still need clarification. Our previous study demonstrated that in response to infection with Gram-negative bacteria, THP-1 macrophages produce PGE2, critical for IL-1β increase (13). PGE2 is a critical immunomodulatory lipid, and for this reason, its synthesis and metabolism are tightly controlled in the cell (64). We showed that PGE2 leads to macrophage polarization toward a proinflammatory M1 phenotype in the infection with Y. enterocolitica and *S.* Typhimurium (13). This regulatory loop that controls IL-1β production via PGE2 might be an important mechanism controlling the output of inflammasome activation in *Yersinia* infection. However, the roles of PGE2 in bacterial infection and pathogen clearance are poorly understood. For instance, while most Gram-negative bacteria increase PGE2 production in the host (8, 9, 13, 21, 22), we have previously shown that Y. enterocolitica restricts the synthesis of this eicosanoid (13), but the effectors involved in this mechanism have not yet been shown. Our present study demonstrated that PGE2 synthesis inhibition is also present in Y. pseudotuberculosis infection, and we further demonstrated that YopJ is one of the effectors used by this bacterium to restrict PGE2 synthesis. We also confirmed previous studies (41) showing that YopJ is critical in the downregulation of IL-1β.

**FIG 9:**
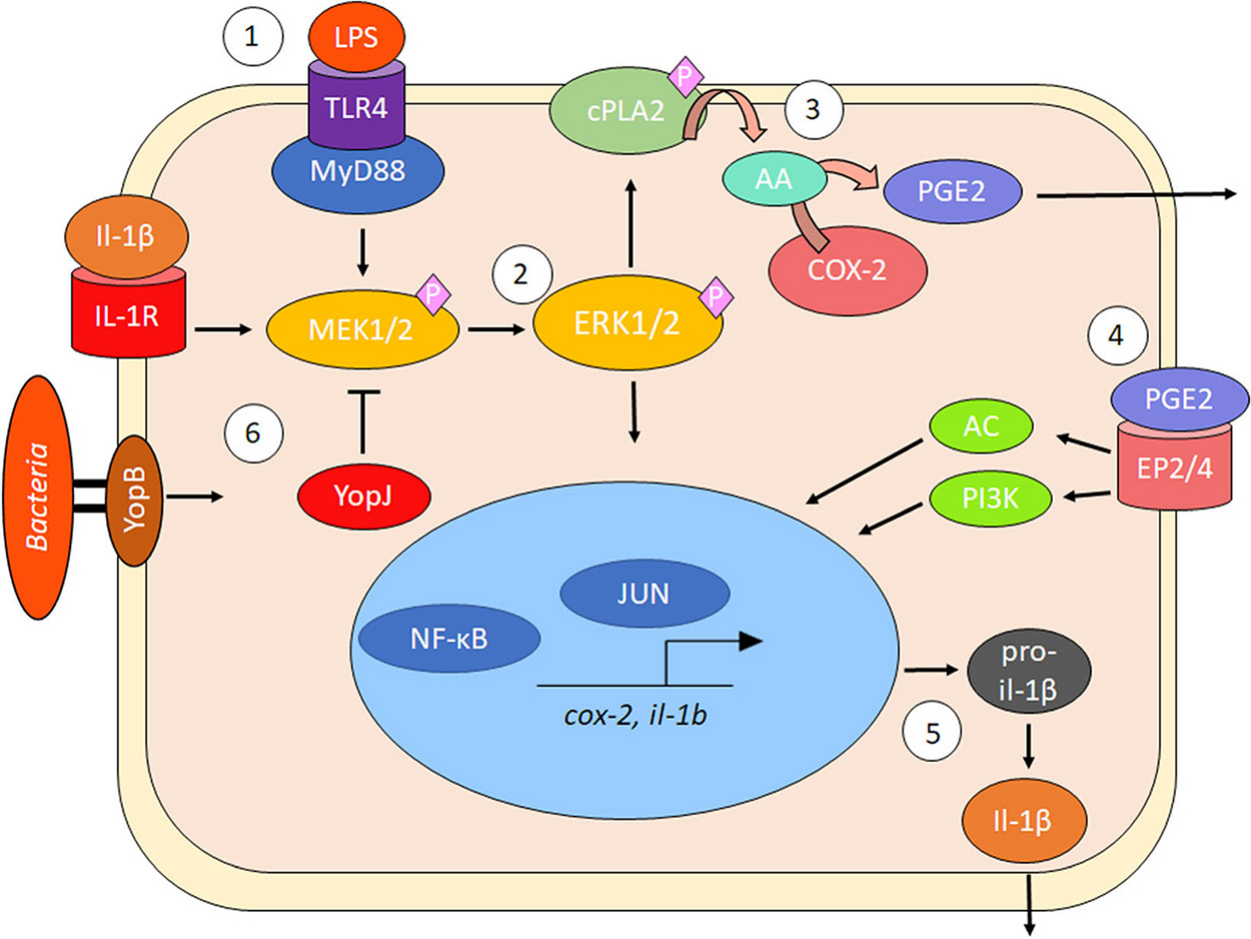
Model of PGE2 and IL-1β biosynthesis in macrophages during Gram-negative bacterial infection. (Step 1) LPS ligand binding to TLR4 induces rapid phosphorylation of host MAPKs, such as MEK1/2, with adaptor protein assistance, including MyD88. (Step 2) MEK1/2 becomes activated and phosphorylates ERK1/2, leading to the activation and migration of transcription factors to the nucleus to induce proinflammatory gene transcription, including upregulation of COX-2 transcripts. (Step 3) Activated ERK1/2 phosphorylates and activates cPLA2 at Ser 515/505 residue, resulting in the liberation of AA from membrane phospholipids and AA’s conversion into PGH2/PGE2 by COX-2. (Step 4) PGE2 is secreted and binds in an autocrine fashion to specific EP2/4 receptors to activate IL-1β transcription by altering cAMP levels or PI3K-induced migration of transcription factors NF-κB and AP-1. In the presence of Gram-negative bacteria, pro-IL-1β is cleaved to mature IL-1β by caspase-1 due to inflammasome activation. (Step 5) IL-1β forms a positive-feedback loop with PGE2 by binding to IL-1R and increasing COX-2 transcription. Y. pseudotuberculosis and S. flexneri modulate inflammasome activation and PGE2 biosynthesis by manipulating host MAPK/ERK-dependent signaling such as T3SS factors YopJ (Y. pseudotuberculosis) or OspB/OspF (S. flexneri).

PGE2 biosynthesis begins with the cleavage of membrane phospholipids, which are converted by the cPLA2 enzyme to arachidonic acid (AA). AA is used as a substrate to produce PGH2 by an enzyme COX-2, which is the primary target of NSAIDs, and PGH2 is later processed into PGE2 by PGE synthase via an isomerization reaction (65, 66). During infection with some Gram-negative bacterial pathogens, including E. coli and Salmonella, bacterial LPS stimulates PGE2 biosynthesis and upregulates COX-2 transcription (9, 21, 51). PGE2 secretion via COX-2 activity is induced by TLR signaling, such as TLR4 or TLR7/8, and impaired in TLR4- or MYD88-deficient macrophages (67, 68). Binding of the lipid A component of LPS to TLR4 (68, 69) results in internalization of the receptor involving the rapid phosphorylation and activation of various MAPKs, such as MEK, JNK, and p38 kinases, which activate proinflammatory cytokine transcription via NF-κB (70). MEK inhibition correlates with COX-2-dependent restriction of PGE2, as shown in Escherichia coli and *S.* Typhimurium infection models (9, 21, 71, 72). Specifically, NF-κB or AP-1 can serve as one way of controlling COX-2 transcription in this process (9, 21, 71, 72), although MEK also controls activation of cPLA2, which converts membrane phospholipids to AA (Fig. 9).

While most Gram-negative bacteria, including Shigella flexneri (see Fig. S4), Salmonella enterica, Escherichia coli, Chlamydia trachomatis, and Legionella pneumophila (8, 9, 13, 21, 22) increase COX-2 transcription and PGE2 synthesis, as demonstrated in this study, Yersinia enterocolitica and *pseudotuberculosis*, downregulate the COX-2-mediated PGE2 biosynthesis. Y. enterocolitica also contains LPS, which should stimulate PGE2 secretion; therefore, the inhibition of COX-2 signaling in this infection was surprising. Interestingly, we also previously discovered that PGE2 was important for the clearance of Y. enterocolitica. Therefore, the fact that the pathogen hampers AA and PGE2 secretion in a pYV-dependent manner indicates that targeting the eicosanoid pathway by *Yersinia* might serve as a pathogenic mechanism. This unique mechanism that inhibits PGE2 synthesis has not been shown in other bacteria. In this study, we were interested in identifying the virulence mechanism that leads to the inhibition of the COX-2 pathway and whether this mechanism leads to the clearance of bacteria. First, we showed that Y. pseudotuberculosis interferes with PGE2 biosynthesis in macrophages (Fig. 1). Heat-killed Y. enterocolitica or Y. pseudotuberculosis also induced secretion of PGE2 from THP-1 macrophages, suggesting that the bacterial factor inhibiting PGE2 is a bioactive component. *Yersinia* uses T3SS to inject effector proteins into the host cell, and the crucial protein that controls this process is YopB translocon protein. YopB participates in forming a pore in the host cell membrane (46), via which *Yersinia* can inject the pathogen effector proteins directly into the host cell. Hence *yopB* controls the translocation of all Yops (73–75). We found that *yopB*-deficient Y. enterocolitica and Y. pseudotuberculosis were unable to inhibit PGE2 biosynthesis from THP-1 macrophages (Fig. 1). Consistently, we discovered that COX-2 protein levels in THP-1 macrophages are upregulated in response to live and heat-killed *Yersinia* infection, as well as positive-control LPS (Fig. 2). We concluded that a protein effector responsible for PGE2 biosynthesis via COX-2 is one of the Yops secreted by Y. enterocolitica and Y. pseudotuberculosis.

We hypothesized that *Yersinia* downregulates PGE2 biosynthesis in response to LPS using YopJ effector, one of six Yops translocated by *Yersinia* to the host cells via T3SS. YopJ inhibits MEK signaling (43), and it is an evolutionarily conserved effector that functions as acetyltransferase (48). The current view is that YopJ effectors work primarily on host substrates via acetylation (76), where Cys172 residue is required for the acetyltransferase properties of YopJ in Y. pseudotuberculosis (43). Our study used Y. pseudotuberculosis strain expressing only catalytically inactive YopJ that harbored a mutation in the Cys172 residue. This mutant strain, along with the wild-type Y. pseudotuberculosis strain, was used to infect host cells and examine the significantly up- and downregulated proteins in host macrophages during the infection. This unbiased approach helped to discover the host pathways targeted by YopJ in host cells. For example, YopJ was revealed to play an essential role in limiting host LPS responses, including repression of EGR1 and JUN proteins, which control transcriptional activation of proinflammatory cytokine production, including IL-1β (Fig. 3F). Pathway analysis of differentially abundant host proteomes predicted that eicosanoid synthesis was downregulated by YopJ in wild-type-infected macrophages compared to a cell infected with a YopJC172A catalytic mutant of this bacterium, and the downregulated molecules included PGE2 and COX-2 (Fig. 3F). These predicted effects of YopJ on the PGE2 biosynthetic pathway via MAPK were confirmed by quantifying COX-2 protein level (Fig. 2 and 6), COX-2 transcripts (Fig. 6), and PGE2 (Fig. 4 and 6). *Yersinia* strains expressing enzymatically inactive YopJ were unable to inhibit COX-2 or PGE2 synthesis. Hence, YopJ clearly downregulates LPS-induced PGE2 biosynthesis in macrophages by targeting COX-2 expression. Moreover, YopJC172A mutant of Y. pseudotuberculosis complemented with wild-type YopJ (48) downregulated COX-2 protein (Fig. 6), as well as COX-2 transcripts in infected THP-1 macrophages. The same phenotype was observed in BMDMs infected with Y. pseudotuberculosis (Fig. 7). In summary, YopJ was demonstrated to serve as a protein that abrogates COX-2 by downregulating its transcription.

The pYV-encoded virulence factor YopJ from Y. pseudotuberculosis and Y. pestis (YopP in Y. enterocolitica) has been shown to suppress IL-1β secretion by downregulating pro-IL-1β synthesis in macrophages via MAPK (13, 40, 41). In response to *Yersinia* infection, MAPKs are rapidly phosphorylated, leading to signal transduction and activation of cytokine transcription. YopJ inactivates several MAPK proteins by transacetylating active site serine and threonine residues, thereby preventing phosphorylation and blockage of signal transduction, including the MAPK-ERK pathway (43). The YopJC172A mutant has been previously documented to be impaired in preventing MEK phosphorylation and signal transduction. To demonstrate the functional importance of the MEK signaling pathway on PGE2 biosynthesis during this Gram-negative infection, we used a chemical inhibitor of MEK signaling during YopJC172A Y. pseudotuberculosis or *ΔyopB* Y. enterocolitica infection of macrophages, which indeed downregulated PGE2 biosynthesis compared to vehicle-treated infected cells (Fig. 4). MEK phosphorylation was sustained in cells infected with YopJC172A and Δ*yopB* strains, compared to wild-type Y. pseudotuberculosis, indicating sustained activation of gene transcription.

Considering that PGE2 biosynthesis is crucial for *il-1b* transcription, IL-1β secretion from THP-1 macrophages correlates with PGE2 secretion. IL-1β secretion is upregulated in response to heat-killed or Δ*yopB* Y. enterocolitica and Y. pseudotuberculosis (13, 36). PGE2 and IL-1β share a positive feedback loop in which PGE2 stimulates the production of IL-1β via upregulation of pro-IL-1β transcription in an EP4-dependent manner (20, 77). Our hypothesis assumed that changes in IL-1β secretion by PGE2 were due to changes in the transcription of *il-1b* since PGE2 is necessary for induction of pro-IL-1β and is in part dependent on EP4 stimulation (13, 17). IL-1β is synthesized in an inactive form in the cell known as pro-IL-1β before being converted to mature and bioactive IL-1β. We found that pro-IL-1β levels in cell lysates are highest in Δ*yopB* and YopJC172A Y. pseudotuberculosis*-*infected macrophages, and they are the lowest in wild-type infection, which correlated with secreted IL-1β levels (Fig. 5). Moreover, *il-1b* mRNA transcripts were increased in YopJC172A Y. pseudotuberculosis*-*infected cells compared to wild-type Y. pseudotuberculosis-infected cells, confirming that YopJ-mediated changes to IL-1β happen on a transcriptional level. Upon complementing the YopJC172A Y. pseudotuberculosis mutant with a wild-type copy of YopJ, the *il-1b* transcription was reduced to wild-type levels (Fig. 5). We consistently noted an increase in IL-1β in cell culture supernatants from BMDMs exposed to heat-killed, Δ*yopB*, or YopJC172A but not wild-type Y. pseudotuberculosis (Fig. 7). The YopJ-regulated PGE2 and IL-1β synthesis are of physiological importance to the host response to infection. Secreted IL-1β acts on local IL-1R receptors to upregulate COX-2 transcription in a MEK-dependent process (17, 57). The feedback loop between COX-2 and IL-1β is of interest since IL-1β plays a vital role in activating immune cells in the immediate vicinity to generate inflammation, neutrophil efferocytosis, and ultimately the phagocytosis and destruction of the invading pathogen (18, 78).

YopJ has several homologs in other bacterial species, and it belongs to the CE clan of cysteine proteases. The members of this family of enzymes have several different enzymatic functions, including ubiquitin-like modifier function, deubiquitinating activity, or Ser/Thr acetyltransferase functions (reviewed in reference 79). Although these enzymes appear to share similar activities, Salmonella’s SseL, Escherichia’s ElaD, *Shigella*’s ShiCE, and *Rickettsia*’s RickCE were shown to be dedicated deubiquitinases, and *Legionella* LegCE, *Yersinia* YopJ, and Salmonella AvrA are dedicated acetyltransferases. At the same time, Chlamydia ChlaDUB1 performs both deubiquitinase and acetyltransferases reactions, using the same catalytic Cys residue (79). One interesting question is whether other CE clan acetyltransferases, such as LegCE and AvrA, encoded by various pathogens but sharing homology with YopJ, also target similar biosynthetic pathways, possibly by affecting different components of these pathways. AvrA, an effector protein of Salmonella, inhibits activation of the JNK pathway activated by this pathogen, which happens in an MKK7-dependent manner (80), while YopJ affects a broader repertoire of MKK proteins compared to AvrA (80). The clear distinction between AvrA and YopJ is that YopJ affects the p38 pathway while AvrA does not. Specifically, AvrA affects only MKK7 (80), specific to the JNK pathway function (81), while YopJ affects MKK3, MKK6, MKK4, and MKK7 (80), where MKK4 activates the p38 pathway (81). Notably, the p38 protein has been previously implicated COX-2 pathway (82). Since AvrA lacks any effect on COX-2 (80), the likely explanation is that the p38 pathway is important for the YopJ function in the COX-2 downregulation. Consistent with this hypothesis, despite the homology of Salmonella’s AvrA to *Yersinia*’s YopJ, AvrA does not complement a YopJ defect in *Yersinia* (83), and AvrA does not affect COX-2 transcripts (80), suggesting a unique function of YopJ. However, there are other bacterial homologs of YopJ worth investigating. Moreover, it is possible that virulence factors that lack homology to YopJ but specifically affect MAPK/ERK signaling affect PGE2 and COX-2 biosynthesis, as is the case with OspB/OspF from *Shigella.* OspB/OspF plays a vital role in limiting PMN migration to the injury site, which may be linked to increased PGE2 secretion and function, considering PGE2 inhibits PMN migration toward macrophages (13). We were able to experimentally demonstrate that knocking out either OspB or OspF resulted in decreased COX-2 transcription during infection with THP-1 macrophages, and a double mutant exhibited a cumulative decrease in COX-2 transcription (see Fig. S2). Therefore, it should be examined whether other pathogens that contain YopJ homologs or affect MAPK/ERK signaling also modulate PGE2 biosynthesis in response to infection.

The examination of differentially regulated host proteins during THP-1 macrophage infection also led to discovering new potential effects of YopJ on the host protein pathways. THP-1 macrophages infected with wild-type or catalytically inactive YopJC172A mutant of Y. pseudotuberculosis had a distinct impact on host proteins. In terms of the comparison between the YopJC172A Y. pseudotuberculosis-infected cells and wild-type strain-infected cells (Table 1), the specific pathways affected by the strain harboring YopJ catalytic mutant were related to sirtuin signaling pathway, NRF2-mediated oxidative stress response, oxidative phosphorylation, Rac signaling, Cdc42 signaling, or mitochondrial dysfunction (Fig. 1E). Among the proteins that were most robustly upregulated in cells infected with YopJC172A-expressing Y. pseudotuberculosis in comparison to wild-type infection, NDRG1 (Q92597) was one of the top hits (FC = 35), followed by JUN (P05412, FC = 23). While JUN was previously shown to be downregulated by active YopJ (48), the downregulation of NDRG1 is a new finding. Moreover, the absence of catalytically active YopJ from cells infected with *Yersinia* upregulated mitochondrial enzymes responsible for oxidative phosphorylation, such as COX17, NDUFA4, NDUFA12, or NDUFS2 (see Fig. S2), some of which might be caused by the downregulation of KDM5 lysine demethylase 5A. KDM5 is an upstream regulator, and its inhibition (or mutation) leads to upregulation of such molecules as NDUFA4, COX17, or NDUFS2 (84), and consequently, the *kdm5* mutants display metabolic defects related to mitochondrial dysfunction (85). These data suggest that YopJ might have a direct or indirect effect on host NAD^+^ output during infection. While the impact of YopJ on oxidative phosphorylation is not yet resolved, it has been previously shown that YopJ expression in Schizosaccharomyces pombe is associated with enhanced sensitivity to oxidative stresses (86).

Finally, this study revealed that EP4 agonist, which acts similarly to PGE2 via EP4 receptor, promotes bacterial clearance after 24 h (Fig. 8), similar to our previous study using PGE2 alone (13). However, the EP4 agonist did not cause a decrease in intracellular Y. pseudotuberculosis that is deficient in YopJ activity after 24 h, suggesting that EP4 agonist only makes a significant difference to bacterial clearance if YopJ is present in the cell, confirming our hypothesis that YopJ-mediated changes to PGE2 biosynthesis have a physiological outcome on infection.

In summary, this study provides several lines of evidence that MEK signaling is critical for PGE2 biosynthesis in host macrophages during Gram-negative infection. We propose that *Yersinia* uses the T3SS effector YopJ to inhibit MEK signal transduction events, which lead to the downregulation of COX-2 transcription (Fig. 9). As a consequence of the enzymatic activity of the YopJ enzyme, pro-IL-1β levels are diminished in host macrophages resulting in a smaller amount of mature IL-1β secretion, a finding consistent with other studies (41). Therefore, we suspect that inhibition of PGE2 by YopJ is one of the mechanisms employed by Y. pseudotuberculosis, but possibly also by other pathogens to attenuate inflammasome-driven IL-1β secretion during pyroptosis. Since other bacterial factors appear to enhance PGE2 biosynthesis, which can be achieved by targeting MAPK signaling, future studies should examine the role of PGE2 during other bacterial infections and investigate whether PGE2 could serve as a potential therapeutic target for bacterial infections.

## MATERIALS AND METHODS

### Cell culture.

THP-1 monocytic cells (ATCC TIB-202; ATCC, USA) were cultured in RPMI 1640 (Gibco/Life Technologies, Inc., USA) supplemented with 10% fetal bovine serum (FBS), 2 mM GlutaMAX (Gibco/Life Technologies), and 100 μg/ml penicillin/streptomycin (Gibco/Life Technologies) in a humidified atmosphere of 5% CO_2_ at 37°C. For activation and differentiation of THP-1 cells into macrophages, phorbol 12-myristate 13-acetate (PMA; Sigma-Aldrich, USA) was used (10 nM), and cells were incubated for 48 h before infection.

The primary bone BMDMs used in this study were isolated and cultured as previously described (87). The BMDMs were isolated from bone marrow flushed from the femurs and tibias of BALB/c mice maintained and euthanized, following the institutional guidelines. The isolated BMDMs were cultured in RPMI 1640 medium supplemented with 10% heat-inactivated FBS, 100 U/ml penicillin, and 100 μg/ml streptomycin. A macrophage colony-stimulating factor (5 ng/ml) was added every 2 days for a week, along with new media to promote macrophage differentiation. After a week, the differentiated macrophages were detached by adding ice-cold phosphate-buffered saline (PBS), followed by incubation at 4°C for 10 min. The macrophages were detached by gently pipetting the PBS of the plates, followed by centrifugation at 200 × *g* for 5 min. For subsequent experiments, the cells were counted, resuspended in sterile BMDM cultivation media, and seeded in sterile 24-well plates.

### Bacterial strains and growth conditions.

Y. enterocolitica 8081 wt (pYV), its isogenic virulence plasmid-cured mutant (8081c) strain, the Ye W22703 wild-type strain, and Ye W22703 Δ*yopB* strain were grown in Luria-Bertani medium (LB) overnight (18 h) at 27°C. The culture was diluted in fresh LB to achieve a final optical density at 600 nm (OD_600_) of 0.05, and such culture was incubated at 27°C for ∼2 h until the OD_600_ reached 0.25. The temperature was then changed to 37°C to activate the T3SS, and bacteria were grown until reaching the OD_600_ reached 0.5, followed by centrifugation at 5,000 × *g*. Bacteria were washed once with prewarmed PBS and resuspended in a cell culture medium for infections. For heat-killed treatments, bacteria were incubated at 65°C for 30 min and allowed to cool to room temperature before infection at an equivalent MOI as viable bacterial infections.

Yersinia pseudotuberculosis strain YopJC172A-pYopJ was created via a conjugation reaction of YopJC172A Y. pseudotuberculosis with SM10l E. coli containing a plasmid with a wild-type copy of YopJ under the control of an IPTG (isopropyl-β-d-thiogalactopyranoside)-inducible promoter, an M-45 C-terminal tag to identify recombinant YopJ in host cell lysates, as well as an ampicillin antibiotic selection marker. Briefly, 1:1, 1:4, and 1:8 ratios of stationary-phase Y. pseudotuberculosis YopJC172A and E. coli pYopJ-M45 were mixed, followed by incubation at 37°C for 2 h. The resulting mixtures were plated on *Yersinia-*selective medium Oxoid containing ampicillin and grown at 27°C for 48 h. Isolated colonies were streaked once more on *Yersinia* selective media Oxoid with ampicillin, and positive transformants were confirmed by PCR.

Yersinia pseudotuberculosis wild-type 32666, Δ*yopB* 32666, YopJC172A, and YopJC172A-pYopJ strains were grown in LB overnight (18 h) at 27°C. The culture was diluted into fresh LB containing 20 mM MgCl_2_ and 20 mM sodium oxalate to a final OD_600_ of 0.1. For induction of wild-type YopJ in the YopJC172A-pYopJ strain, IPTG was added at the indicated final concentrations. Each subculture was incubated at 27°C for 1 h and then changed to 37°C for 2 h to activate the T3SS. Bacteria were centrifuged at 5,000 × *g*, washed with PBS, and resuspended in a cell culture medium for infections.

GFP-containing, fluorescent Y. pseudotuberculosis wild-type, and YopJC172A 32666 strains were generated by electroporation with pON::sfGFP plasmid. To generate sfGFP plasmid, the sfGFP coding sequence was digested out of a donor plasmid (pMJG138) with EcoRI/XbaI. The pON.mCherry plasmid (88) was digested using the same digestion enzymes. The digestion was confirmed by gel electrophoresis resolved on 1% agarose. The DNA fragments were excised from the gel and purified, and the sfGFP insert was ligated into the pON plasmid. The resulting ligation product (pDC41) was transformed into chemically competent DH5α cells by heat shocking, and the colonies expressing green fluorescent protein (GFP) were selected on chloramphenicol plates. This sfGFP plasmid was used for transformation. Briefly, *Yersinia* cultures were grown in 20 ml of LB in a 125-ml flask at 27°C for 3 h. Mid-log-phase bacteria were centrifuged at 5,000 × *g* for 5 min, washed, and resuspended in ice-cold 10% glycerol. This washing process was repeated for a total of four washes. Electrocompetent ells were concentrated 100× and used immediately for electroporation in a Bio-Rad Gene Pulser. An aliquot containing a 1 μl of plasmid was used for each 50-μl reaction of bacterial cells. Bacteria were recovered at 37°C for an hour, followed by plating on chloramphenicol-containing LB plates.

Yersinia enterocolitica 8081 wt (pYV), its isogenic virulence plasmid-cured mutant (8081c), Yersinia pseudotuberculosis wild-type 32666, Δ*yopB* 32666, and YopJC172A strains were generous gifts from James Bliska, while Olaf Schneewind provided the Ye W22703 wild-type strain and the Δ*yopB* strain. The YopJC172A-pYopJ strain was generated via conjugation with E. coli containing a pMMB67HE plasmid encoding YopJM45 under the control of a tac promoter, Ampr (an alternative name is pLP17, as described previously).

Shigella flexneri 2a 2457T wt, *ΔospF*, *ΔospB*, and *ΔospF ΔospB* strains were generous gifts from Anthony Maurelli (73). All *Shigella* strains were first streaked on Congo red agar and grown at 37°C to confirm the presence or absence of the virulence plasmid (88).

All strains used in this study are listed in Table 2.

**TABLE 2:** Strains used in this study

Strain	Phenotype/genotype	Reference
Y. enterocolitica		
W22703 wt	wt (wild type)	91
W22703 Δ*yopB*	Δ*yopB*	92
		
Y. pseudotuberculosis		
32777 wt serotype O3	wt	93
32777 serotype O3 Δ*yopB*	Δ*yopB*	94
32777 serotype O3 YopJC172A	YopJC172A, catalytic inactivation of YopJ	95
32777 serotype O3 YopJC172A pYopJ-M45	Plasmid expressing YopJ under IPTG-inducible promoter	95
32777 wt serotype O3 sfGFP	wt, sfGFP fluorescent tag	This study
32777 serotype O3 YopJC172A sfGFP	YopJC172A, catalytic inactivation of YopJ, sfGFP fluorescent tag	This study
		
S. flexneri		
2a 2457T wt	wt	96
2a 2457T Δ*ospB*	Δ*ospB*	97
2a 2457T Δ*ospF*	Δ*ospF*	73
2a 2457T Δ*ospF* Δ*ospB*	Δ*ospF* Δ*ospB*	97
		
E. coli SM10/λ*pir* pYopJ-M45	Plasmid expressing YopJ under IPTG-inducible promoter	48

### Infections and LPS treatment.

THP-1-derived macrophages differentiated for 48 h before infection as described above were washed with PBS and incubated in RPMI media containing 10% FBS and no antibiotics for 60 min before infection. Bacteria were washed with PBS, resuspended in RPMI media containing 10% FBS, and used to infect cells (MOI of 50:1) for the times indicated in figures; the cell culture supernatant and cells were then collected and centrifuged once to remove bacteria, and the supernatant was recovered. For Shigella flexneri infections, overnight cultures of S. flexneri were grown in LB for 18 h at 37°C and were diluted to an OD_600_ of 0.05 the day of the infection. Each strain was grown until the exponential phase (OD_600_ of 0.5) and centrifuged at 5,000 × *g* for 5 min to remove any residual growth media. The bacterial cells were then resuspended in RPMI media at an MOI of 15:1 for infecting THP-1 macrophages. Otherwise, macrophages were treated as indicated above. Alternatively, THP-1 macrophages were stimulated with 10 μg/ml Salmonella enterica serovar Typhimurium lipopolysaccharide (LPS; Sigma-Aldrich) or Yersinia pseudotuberculosis LPS obtained from Y. pseudotuberculosis grown at 37°C in LB as previously described (60) for the times displayed in the figures. The uninfected control cells were treated in the same way as the infected cells, but no bacteria or LPS was added.

### Mass spectrometric analysis.

THP-1 cells were infected for 2 h with Y. pseudotuberculosis wild-type or YopJC172A at an MOI of 50:1 as described above. Uninfected cells treated in the same manner as the infected cells were used as a control. Cells were collected by scraping into cold PBS buffer and centrifuged at 500 × *g* for 10 min at 4°C. The cell pellets were then stored at −80°C until lysis. The cells were lysed in radioimmunoprecipitation assay (RIPA) buffer supplemented with pierce cocktail protease inhibitor tablet for 30 min, and then the samples were centrifuged at 18,000 × *g* for 10 min at 4°C to separate the soluble protein fraction. Protein concentration was determined by bicinchoninic acid (BCA) assay, and samples normalized to an equal amount of protein (30 μg per sample) were prepared to contain an equal volume of lysis buffer. Three independent biological replicates were used in this experiment. Proteins were then separated by SDS-PAGE as a cleanup strategy, and an entire lane was excised by scalpel, which was subsequently diced into 1-mm^2^ cubes. These protein samples were subjected to in-gel trypsin digestion performed precisely as we did previously (89), followed by C_18_ ZilTip purification (Waters).

The mass spectrometric analysis was performed by using 250-mm ultrahigh-performance liquid chromatography coupled to an Orbitrap Fusion mass spectrometer (Thermo Scientific). The liquid chromatography was performed using the Thermo EASY nano-LC system. A 20-mm C_18_ precolumn (Thermo Scientific) was used before the samples were separated by a reversed-phase C_18_ analytical column (100 Å pore; Thermo Scientific, Acclaim PepMap 100 C_18_ LC column). The samples were purified over a 105-min acetonitrile gradient, followed by a 5-min column wash and a 10-min column equilibration. The LC system was interfaced in-line with an Orbitrap Fusion mass spectrometer (Thermo Scientific), where the MS data were acquired at 120,000 resolution by Orbitrap detector (scan range, 350 to 1,800 *m/z*). The ions were isolated by a quadrupole during MS/MS analysis when the most intense ions were prioritized, and ions were injected for all available parallelizable time. The masses of precursor ions that were selected for fragmentation were then excluded for 15 s to minimize the acquisition of data for the identical ions. Ion fragmentation was done by using collision-induced dissociation at a collision energy of 35%; the activation time was 10 min, and the AGC target was 10,000. The ion fragments that resulted from these MS/MS data were detected by the ion trap.

Tandem mass spectra were extracted, charge state deconvoluted, and deisotoped by Proteome Discoverer version 2.4 (Thermo Scientific). The MS/MS samples were analyzed by using Sequest (Thermo Fisher Scientific, San Jose, CA; version IseNode in Proteome Discoverer 2.4.1.15) and X! Tandem (The GPM; version X! Tandem Alanine 2017.2.1.4). Sequest was programmed to search FASTA human and Yersinia pseudotuberculosis UniProt database containing a common list of contaminants database (24,311 entries) assuming the digestion enzyme trypsin. X! Tandem was set up to search a reverse concatenated subset of the same database set. Sequest and X! Tandem were searched with a fragment ion mass tolerance of 0.60 Da and a parent ion tolerance of 10.0 ppm. Carbamidomethyl of cysteine was specified in Sequest and X! Tandem as a fixed modification. The following variable modifications were also searched: Met-loss of methionine, met-loss+Acetyl of methionine, oxidation of methionine, and acetylation of the N terminus.

Scaffold (version Scaffold 4.11.0; Proteome Software, Inc., Portland, OR) was a software that was used to validate MS/MS-based peptide and protein identifications. Peptide identifications were accepted if they could be established at greater than 95.0% probability. Peptide probabilities in X! Tandem were assigned by the Scaffold Local FDR algorithm, while the Peptide Prophet algorithm assigned peptide probabilities in Sequest with Scaffold delta-mass correction. Protein identifications were accepted if they could be established at greater than 95.0% probability and contained at least two identified peptides. The Protein Prophet algorithm was used to assign protein probabilities. Proteins that contained similar peptides and could not be differentiated based on MS/MS analysis alone were grouped. The reported peptide false discovery rate (FDR) was 0.02%, and the protein FDR was 0.4%. The weighted spectral count was used for protein quantification, where the spectral counts of peptides were normalized to the total spectral count in each run, and a minimum value of 0.1 was used for samples with missing values to enable the fold change calculations. The fold change was calculated from the spectral count of proteins from control cells or YopJC172A Y. pseudotuberculosis mutant cells versus wild-type Y. pseudotuberculosis-infected cells and wild-type cells versus control (uninfected) cells (see Tables S1 and S2). A Student *t* test was used to calculate statistical significance and a *P* value of <0.05 for the indicated proteins with statistically significant changes.

Morpheus software was used to visualize the protein abundance using a heat map and hierarchical clustering of samples (https://software.broadinstitute.org/morpheus/). Venn diagrams, including the number of proteins in each sample category, were constructed to include the protein numbers for protein hits that appeared in each sample.

### Pathway, function, and network analysis.

Ingenuity Pathway Analysis software (Qiagen) was used for network analysis of proteins with altered abundance. Canonical pathways and bio-functions of proteins were analyzed by the right-tailed Fisher exact test and Hochberg-Bonferroni multiple testing corrections. The significance was calculated to associate protein identification with the canonical pathway and ensure that the possibility that these associations do not happen by random chance alone; the –log of this *P* value is shown on the *x*-axis. For canonical pathway analysis, the number of genes in a pathway identified in the data set was displayed for both proteins, which were both upregulated (red) and downregulated (green) in infected cells or YopJC172A Y. pseudotuberculosis mutant cells versus wild-type Y. pseudotuberculosis-infected cells. The total number of known genes was displayed in the right part of the graph. The top protein networks were identified and overlaid with the most significant pathways. The upstream network analysis was also performed, and the regulators predicted to be activated or inhibited were identified based on z-scores higher than +2/–2.

### Western blot analysis.

THP-1 cell pellets were lysed in RIPA buffer supplemented with protease and phosphatase inhibitors (Roche, USA) on ice for 0.5 h and spun at 16,000 × *g* for 0.5 h, and the supernatant was collected. Protein concentration was quantified using a BCA protein assay kit (Thermo Fisher Scientific). Protein samples were separated by using 4 to 12% gradient SDS-PAGE and transferred onto a polyvinylidene difluoride membrane (Bio-Rad, USA), where the transfer buffer contained 15% methanol, 25 mM Tris, and 192 mM glycine. Membranes were blocked with 5% nonfat milk in TBS containing 0.1% Tween, and proteins of interest were detected by immunodetection with the appropriate antibodies and enhanced chemiluminescence. The following antibodies were used in this study: COX-2 antibody AF4198 from R&D Systems (1:300 dilution), IL-1β antibody SC-7884 from Santa Cruz (1:1,000), β-actin antibody SC-47778 from Santa Cruz (1:1,000), MEK1/2 antibody 8727 from Cell Signaling (1:3,000), Phospho-MEK1/2 antibody MA5-15016 from Invitrogen (1:1,000), M-45 antibody from the Bliska Lab (1:3,000), goat anti-mouse antibody 31430 from Thermo Scientific (1:3,000), goat anti-rabbit antibody 31460 from Thermo Scientific (1:3,000), and donkey anti-goat antibody HAF017 from R&D Systems (1:3,000). All secondary antibodies were horseradish peroxidase conjugated and prepared fresh for each Western blot. Primary antibodies were stored in 0.5% nonfat milk in TBS containing 0.1% Tween and 0.02% sodium azide at 4°C. In the case of phospho-MEK1/2 antibodies, bovine serum albumin (BSA) was used in place of nonfat milk. To compare the abundance of phosphorylated MEK1/2 compared to overall MEK1/2 abundance, the same blot was used and was first probed with phospho-MEK1/2 antibody and then stripped in Restore Plus stripping buffer for 5 min before blocking with 5% BSA for 1 h. The blot was then probed for MEK1/2 and β-actin (loading control).

### MAPK inhibitor treatment.

A total of 200,000 THP-1 cells were seeded in a 24-well plate and differentiated into macrophages with 10 nM PMA for 48 h. One hour before infection, the cells were washed, and complete cell culture medium lacking antibiotics was added; this was supplemented with 10 μM MAPK/ERK antagonist PD184161 from Cayman Chemicals (USA) or an appropriate vehicle control at the same volumetric concentration. THP-1 macrophages were then infected with an MOI of 50:1 of wild-type and Δ*yopB* Y. enterocolitica for 2 h, whereas the resulting supernatant was collected, spun at 500 × *g* to remove cell debris, and analyzed via commercial monoclonal PGE2 ELISA from Cayman Chemicals. Infections with wild-type and YopJC172A Y. pseudotuberculosis were performed at the same time as the Y. enterocolitica infections at the same MOI of 50:1 for 2 h. Cells from all treatments were processed in the same way to improve the consistency of the results.

### Transcript analysis by RT-qPCR.

The total RNA from untreated or infected THP-1 macrophages was extracted using a Qiagen RNeasy Mini Plus extraction kit, followed by cDNA generation using the Verso cDNA synthesis kit (Thermo Fisher) with randomized hexamers. The expression of genes COX-2 and pro-IL-1β were measured using a two-step quantitative real-time PCR (RT-qPCR), which was performed using SYBRGreen reagents (Bio-Rad) on the Stratagene MXP3005. Gene expression was normalized to the housekeeping gene GAPDH first and then compared to the vehicle control treatment expressed as fold change estimated using the ΔΔ*C_T_* method, and the statistical significance was reported as previously described (13, 90). GAPDH was chosen out of three housekeeping genes, including HPRT1 and RPL37A, for comparisons due to GAPDH mRNA level consistency across treatment samples and proximity to *C_q_* values of target templates COX-2 and IL-1β. Primers used for the study have been based on prior work (13).

### Quantification of PGE2 by ELISA.

Prostaglandin E2 ELISA based on monoclonal antibody detection against PGE2 (catalog no. 514010; Cayman Chemicals) was performed to quantify PGE2 present in cell culture supernatants, which were prepared by centrifuging cell supernatants at 500 × *g* for 5 min to remove cell debris; the resulting supernatant was probed using a commercial ELISA kit. The ELISA kit was used per the manufacturer’s recommendations, and the plate was read by using a Cytation3 imaging plate reader (BioTek, USA).

### Fluorescence assay to measure intracellular survival of bacteria.

30,000 THP-1-derived macrophages were seeded on a black 96-well plate and treated with EP4 agonist L-902688 (Cayman Chemicals) or vehicle control (0.01% [vol/vol] DMSO) in RPMI lacking antibiotics for 30 min prior to infection with GFP-expressing wild-type and YopJC172A Y. pseudotuberculosis at an MOI of 15:1 for 1 h. At 1 hpi, media were removed, and cells were washed twice with PBS to remove extracellular bacteria. RPMI media lacking antibiotics but supplemented with gentamicin (100 μg/ml) and with or without 10 μM EP4 agonist L-902,688 or 10 μM JUN inhibitor T-5224 were added to the cells, and the cells were incubated further for another hour. At 2 hpi, the media were removed again, and the cells were washed twice with PBS and resuspended in media containing a lower concentration of gentamicin (10 μg/ml) for the remainder of the infection. For bacterial CFU counts, the cells were lysed with 0.1% Triton X-100, serially diluted in sterile PBS, plated on LB, and allowed to grow overnight at 37°C. Colonies were counted and calculated as CFU/ml. For fluorescence imaging, the THP-1 cells were stained with Hoechst stain at the indicated times (2 and 24 hpi). The cells were then washed with PBS, fixed in 4% PFA, and visualized by Cytation 5 (BioTek).

### Statistical analysis.

Statistical analysis was performed by using GraphPad Prism. A Student *t* test was used with a 95% confidence interval (*P* < 0.05). Alternatively, ANOVA tests in conjunction with Tukey’s multiple-comparison tests were also used, as indicated in the figures. Densitometry analysis of Western blots was performed in ImageJ.

### Institutional safety procedures.

Accidental exposure to pathogenic bacteria here described can cause gastroenteritis and enterocolitis. Standard BSL2 practices were followed, and personnel was carefully advised about biohazards and ways to minimize the chances of exposure. The study was completed by following the standard operating procedures outlined for this project.

### Data availability.

The proteomics data, including raw data files, peak list files, and processed files, are available via Mendeley Data (10.17632/xfwf2nkfg4.1; https://data.mendeley.com/datasets/xfwf2nkfg4/draft?a=36113178-dff3-4a65-8f7a-fa001c9d8e68).
