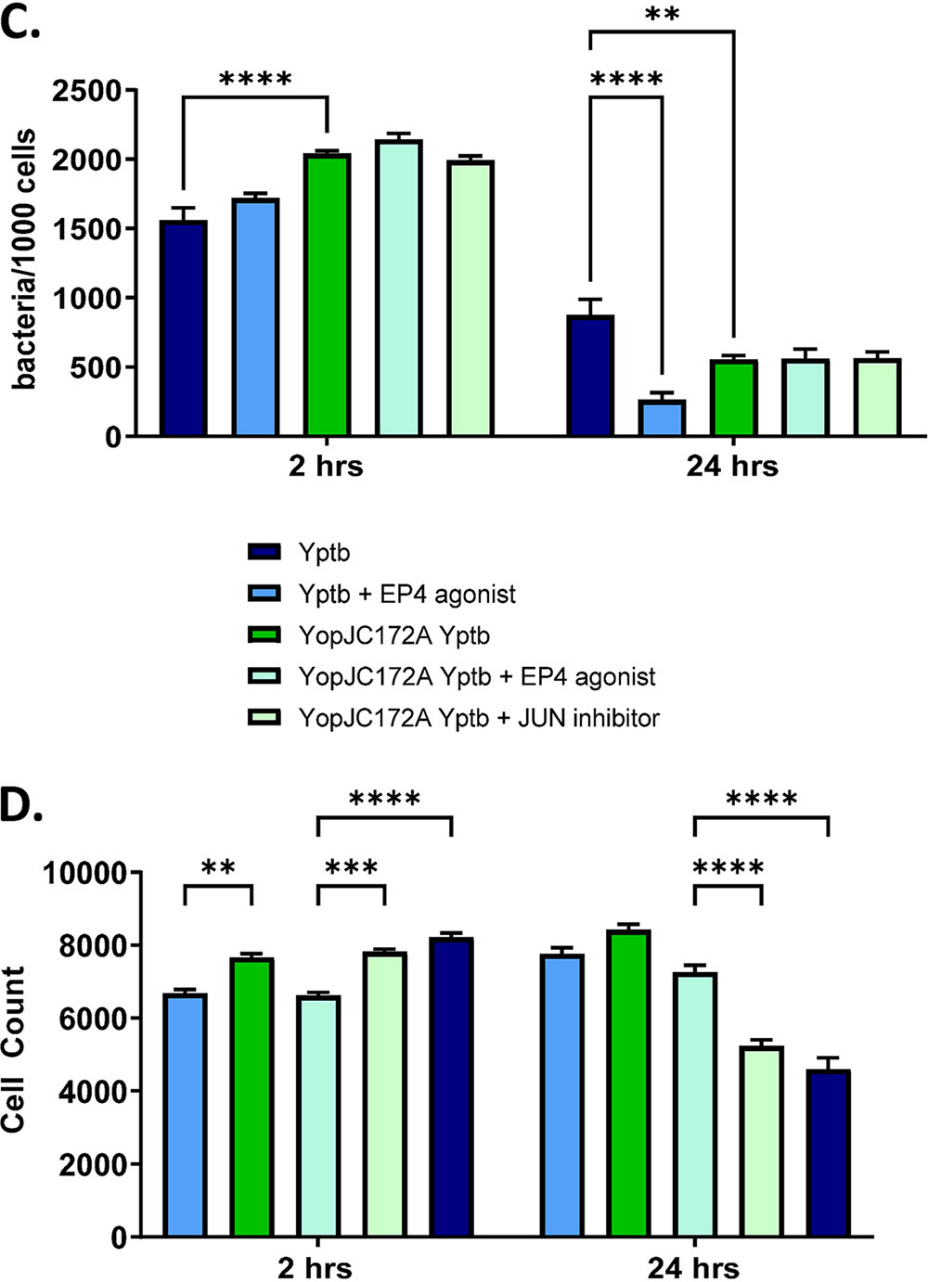# Erratum for Sheppe et al., “Yersinia pseudotuberculosis YopJ Limits Macrophage Response by Downregulating COX-2-Mediated Biosynthesis of PGE2 in a MAPK/ERK-Dependent Manner”

**DOI:** 10.1128/spectrum.03997-22

**Published:** 2023-04-06

**Authors:** Austin E. F. Sheppe, John Santelices, Daniel M. Czyz, Mariola J. Ferraro

## ERRATUM

Volume 9, no. 1, e00496-21, 2021, https://doi.org/10.1128/Spectrum.00496-21. Figure 3E and F and Fig. 8C and D were omitted from the HTML version, although they do appear in the PDF version. The missing panels should appear as shown below.

**Figure 1:**
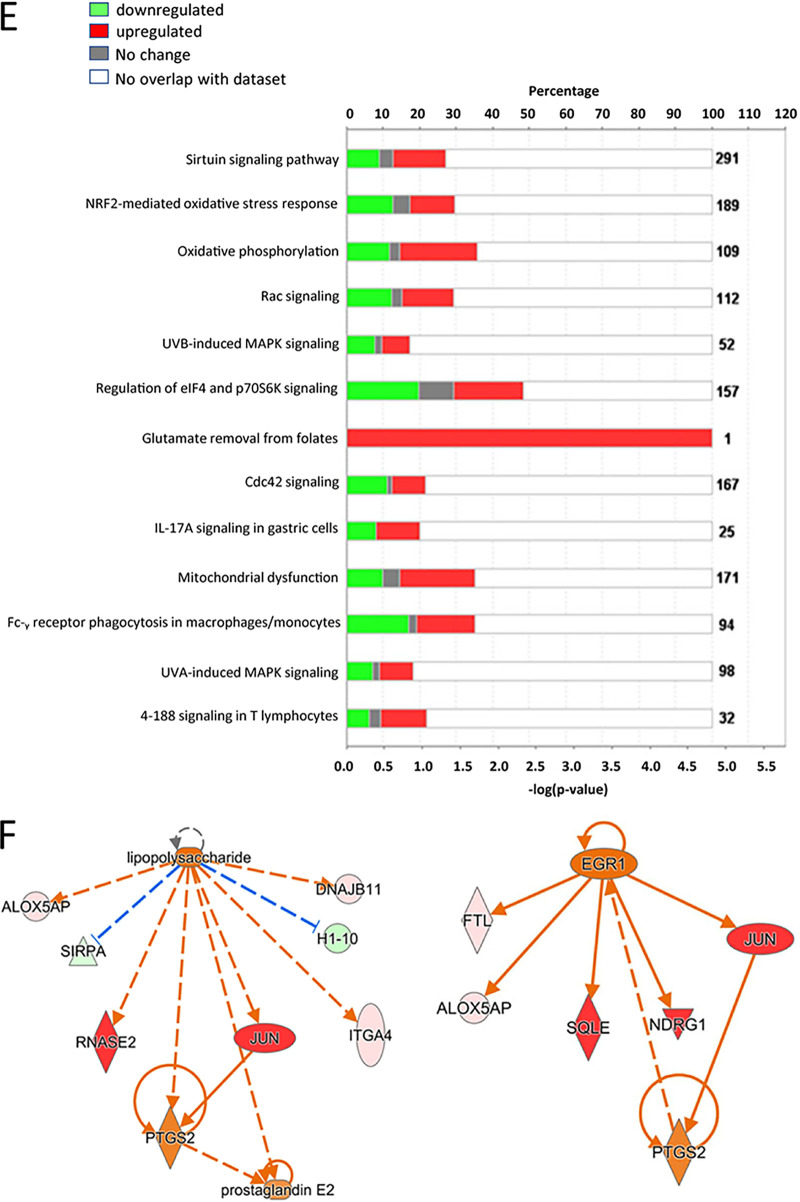


**Figure 2:**