# Supplementary material for: Yersinia pseudotuberculosis YopJ Limits Macrophage Response by Downregulating COX-2-Mediated Biosynthesis of PGE2 in a MAPK/ERK-Dependent Manner
Source: Microbiol Spectr. 2021 Jul 28;9(1):10.1128/spectrum.00496-21. doi: 10.1128/spectrum.00496-21 (PMC8552654; doi:10.1128/spectrum.00496-21)

Supplementary material:

Fig. S1. Volcano plot showing graphical representation of quantitative proteomics data. Proteins were ranked in a volcano plot according to their statistical P-value (y-axis) and relative abundance ratio (log2 fold change) between YopJC172A *Y. pseudotuberculosis*-infected and wild-type *Y. pseudotuberculosis*-infected samples (x-axis).

Fig. S2. (A, B). Oxidative phosphorylation as one of top canonical pathways in proteins differentially abundant in YopJC172A mutant of *Y. pseudotuberculosis*-infected macrophages in comparison to wild-type *Y. pseudotuberculosis*. The graphs represent down-regulated proteins (green), upregulated proteins (red), and proteins with unchanged abundance (gray). **(C).** The upstream regulator identified in YopJC172A *Y. pseudotuberculosis*-infected macrophages in comparison to wild-type *Y. pseudotuberculosis*. The graphs represent down-regulated proteins (green), upregulated proteins (red), while the molecules shown in orange are predicted to be activated, and orange lines indicate direct inhibition.

Fig. S3. The YopJ function in modulating PGE2 biosynthesis in *Y. pseudotuberculosis*-infected THP-1 macrophages. PMA-differentiated THP-1 macrophages were infected with live wild-type *Y. pseudotuberculosis* (Yptb), Δ*yopB,* YopJC172A, or YopJC172A mutant expressing YopJ (YopJ-M45) at an MOI of 50:1 for two hours. YopJC172A + pYopJ-M45 strain was grown in the presence of 0.1 mM IPTG before infection. Alternatively, cells were treated with heat-killed Δ*yopB Y. pseudotuberculosis*, or LPS from *Salmonella* (ST LPS), or *Y. pseudotuberculosis* (Yptb LPS). PGE2 concentration in cell culture supernatants was measured using monoclonal ELISA. One-way ANOVA and Tukey's post hoc test were used to calculate significance (n=3). p-values were indicated as follows: $*p \le 0.05$; $**p \le 0.01$; $***p \le 0.001$; $****p \le 0.0001$.

Fig. S4. OspB/OspF from *Shigella flexneri* 2457T 2a supports COX-2 mRNA transcription in THP-1-derived human macrophages in response to infection. THP-1 macrophages were infected with wild-type, virulence plasmid deficient, Δ*ospB*, Δ*ospF*, or Δ*ospB* Δ*ospF Shigella flexneri* 2a at an MOI of 15:1 for 2 hours. 2 hours post-infection cell pellets were isolated and total RNA was collected via Qiagen RNeasy kit. RT-PCR analysis was performed on COX-2

transcripts and normalized to GAPDH reference gene before comparing to uninfected vehicle control. Statistical significance was calculated using the student's T-test, and p-values were indicated as follows: $*p \leq 0.05$; $**p \leq 0.01$; $***p \leq 0.001$; $****p \leq 0.0001$. The data shown are representative of three experiments.

Table S1. **Shotgun proteomics and pathway analysis of THP-1 macrophages infected with wild-type or YopJC172A mutant *Y. pseudotuberculosis:* Human proteins identified in YopJC172A *Yersinia pseudotuberculosis* -infected THP-1 macrophages in comparison to wild-type *Yersinia* infected macrophages (tab 1), or wild-type *Yersinia* infected macrophages in comparison to uninfected control cells (tab 2).** The table includes a gene symbol, Entrez gene name, Protein accession number, experimental p-value calculated by using t-test, and experimental fold change value calculated based on the normalized and weighted spectral count. Protein location, molecule type, and Entrez Gene ID for Human genes are also shown.

Table S2. **Shotgun proteomics and pathway analysis of THP-1 macrophages infected with wild-type or YopJC172A mutant *Y. pseudotuberculosis: Yersinia* proteins in YopJC172A *Yersinia pseudotuberculosis*-infected THP-1 macrophages in comparison to wild-type *Yersinia* infected macrophages.** The protein name, Uniprot accession number, and molecular weight of identified proteins are shown. Moreover, experimental p-value calculated by using t-test, and experimental fold change value calculated based on the normalized and weighted spectral count are shown. Spectral counts for each biological replicate are also shown.

Supplementary Figure 1

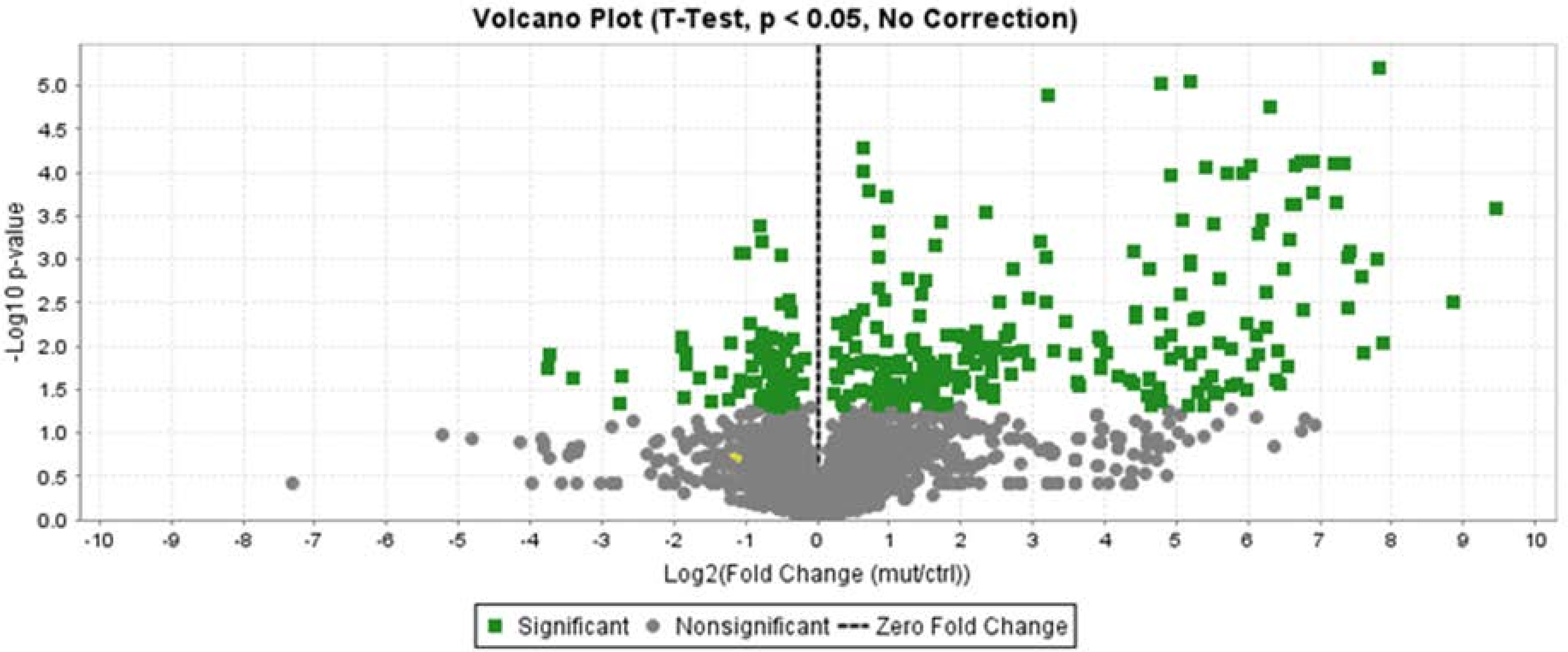

Supplementary Figure 2

A

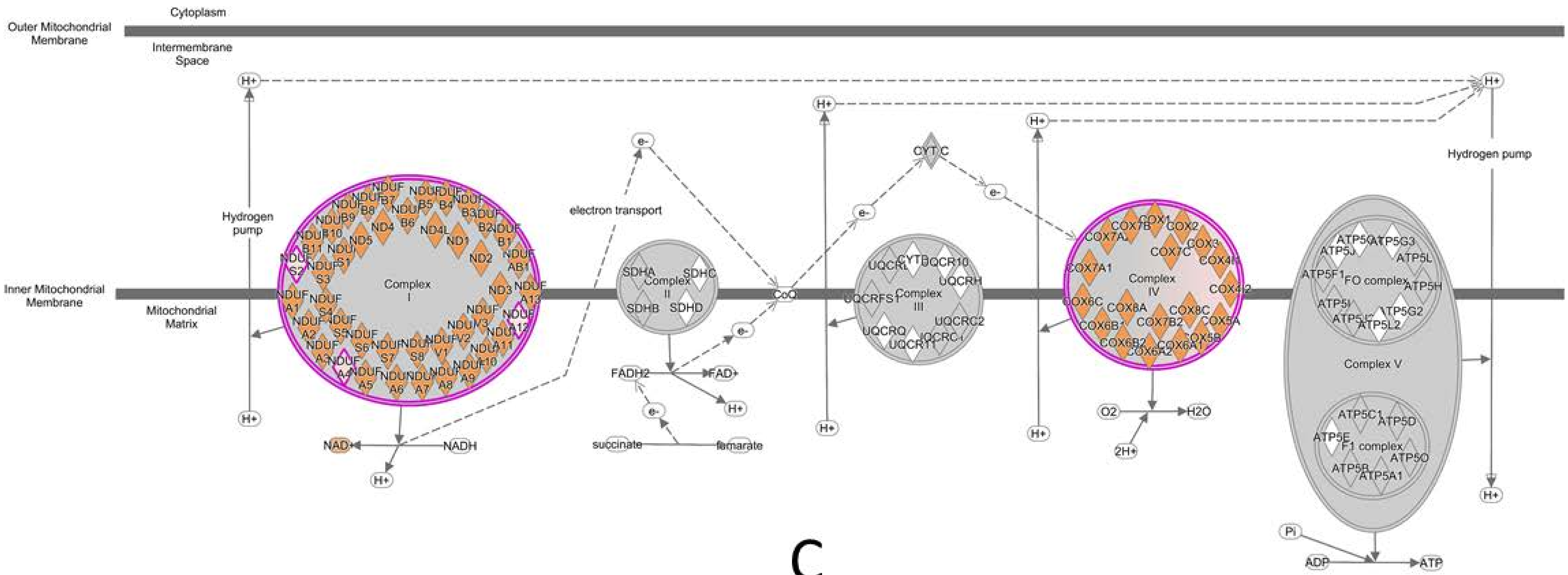

B

Symbol	Entrez Gene Name	Gene Accessio	Expr p-value	Fold Change
COX17	cytochrome c oxidase copper chaperone COX17	Q14061	0.036	3.5
NDUFA4	NDUFA4 mitochondrial complex associated	O00483	0.016	4.1
NDUFA12	NADH:ubiquinone oxidoreductase subunit A12	Q9UI09	0.042	1.8
NDUFS2	NADH:ubiquinone oxidoreductase core subunit S2	O75306	0.04	3.7

C

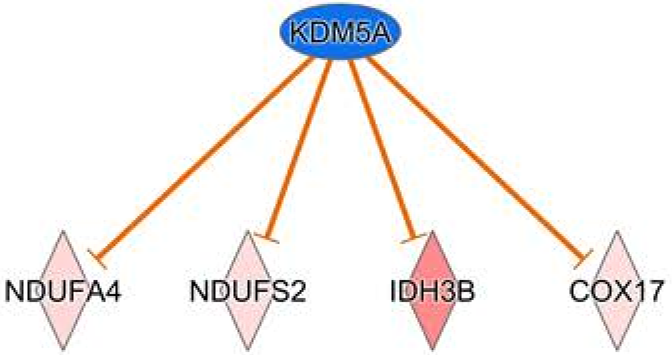

Supplementary Figure 3

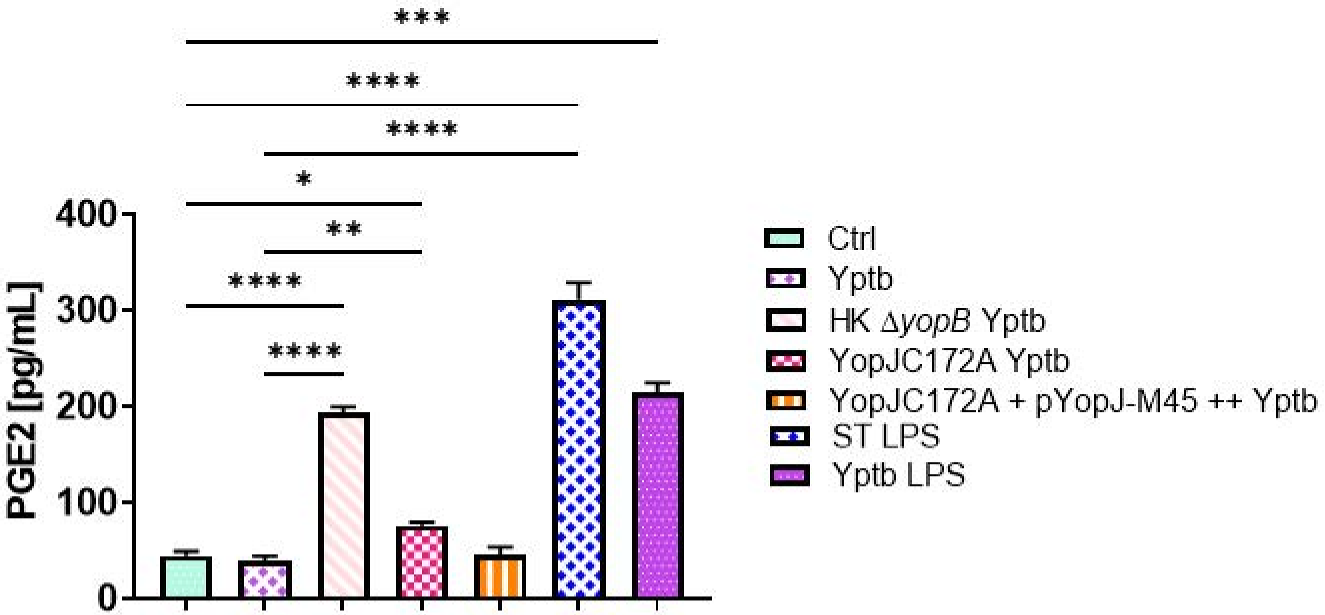

Supplementary Figure 4

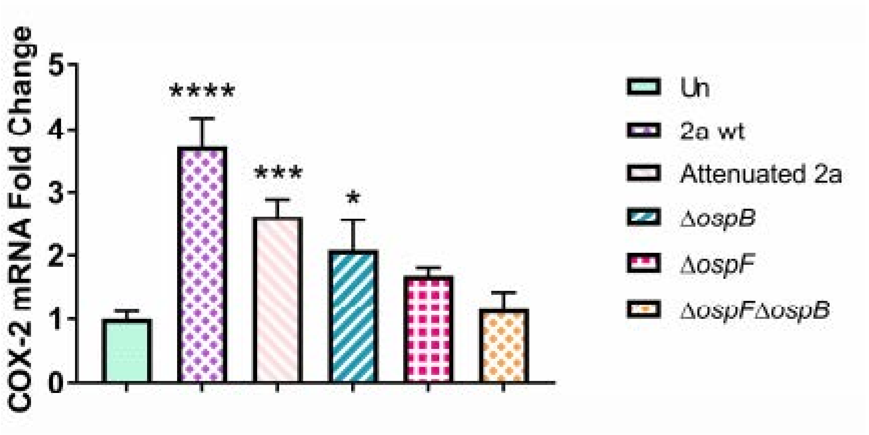

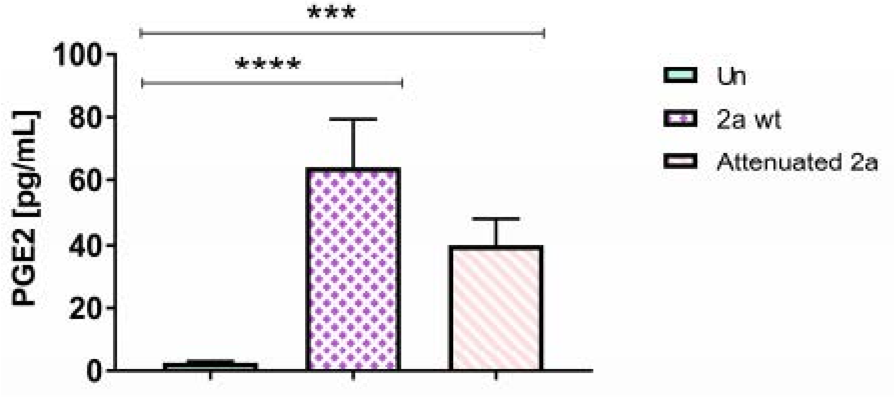

Supplement: SUPPLEMENTAL FILE 3 — Supplemental material. Download SPECTRUM00496-21_Supp_3_seq3.pdf, PDF file, 0.3 MB. [file spectrum00496-21_supp_3_seq3.pdf]
